# Millennia of Metacommunity Diversification and Homogenization Captured by Sedimentary Ancient DNA

**DOI:** 10.1111/ele.70218

**Published:** 2025-09-22

**Authors:** Dilli P. Rijal, Kari Anne Bråthen, Antony G. Brown, Peter D. Heintzman, Inger G. Alsos, Nigel G. Yoccoz

**Affiliations:** ^1^ The Arctic University Museum of Norway, UiT—The Arctic University of Norway Tromsø Norway; ^2^ Department of Arctic and Marine Biology UiT—The Arctic University of Norway Tromsø Norway; ^3^ Centre for Palaeogenetics Stockholm Sweden; ^4^ Department of Geological Sciences Stockholm University Stockholm Sweden

**Keywords:** beta diversity, diversification, GAM, homogenisation, immigration, metacommunity, *sed*aDNA, taxonomic richness, zeta diversity

## Abstract

Alpha (α), beta (𝛽), gamma (𝛾) and zeta (𝛇) diversity metrics are complementary in their information, yet insight from this complementarity has yet to be explored. Here we use postglacial lake sediments for reconstructing plant metacommunity diversity patterns using all four metrics. Based on sedimentary ancient DNA data, we find that the metacommunity both diversified (𝛽_spatial_) and homogenised (𝛇) over millennia of ecosystem development, alongside rising taxon richness at both community (α) and metacommunity (𝛾) level. In contrast temporal turnover of taxa (𝛽_temporal_) declined, both at the community and metacommunity level. With taxon appearances exceeding disappearances this suggests the co‐existence of taxa in the communities increased. However, the shared taxa in the metacommunity (𝛇) showed a continuously high temporal turnover, suggesting the taxa contributing to the metacommunity homogenisation were largely transient. That communities homogenised but remained distinctively different over millennia highlights the importance of individual communities in sustaining metacommunity biodiversity.

## Graphical abstract

The study examines 13,000 years of plant metacommunity change using sedimentary ancient DNA and multiple diversity metrics (α, 𝛽, 𝛾, 𝛇). It finds increasing taxon richness and local coexistence, with long‐term diversification and transient homogenisation. Despite regional trends, high turnover of shared taxa among communities persisted, underscoring the role of individual communities in maintaining biodiversity.

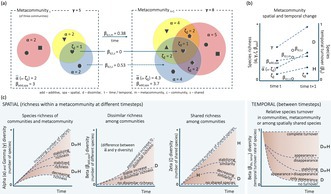

## 1 Introduction

The functioning of ecological communities is linked to their biodiversity (Hooper et al. 2005; Loreau et al. 2022), including the stability of their aggregate ecosystem properties (Loreau et al. 2021). However, it is the intrinsic character of ecological communities to continuously change their biodiversity (Darwin 1859; MacArthur and Wilson 1967; Magurran et al. 2019; Vellend 2010). This implies that legacies from former compositions can have consequences for biodiversity at a later stage through priority effects or niche construction (Jackson and Blois 2015; Odling‐Smee et al. 2013; Stroud et al. 2024; Svenning et al. 2015). Furthermore, ecological community biodiversity change does not occur in isolation, as changes in one community may affect the biodiversity and ecosystem functioning in a neighbouring community (Leibold et al. 2004; Mori et al. 2018). Hence, documenting spatio‐temporal change in the biodiversity of ecological communities is essential for predicting changes in ecosystem functioning.

Biotic homogenisation, a process whereby genetic, taxonomic or functional similarities of regional biotas increase over time (Blowes et al. 2024; *sensu* Olden and Rooney 2006), is a biodiversity change related to the loss of biodiversity in our time (Blowes et al. 2024; Bråthen et al. 2024; Daru et al. 2021; Mori et al. 2018; Staude et al. 2022; Yang et al. 2021). However, biotic homogenisation has also been found in regions where species richness has been increasing (Finderup Nielsen et al. 2019) or has not changed (Bråthen et al. 2024), and species loss has been documented in the absence of biotic homogenisation (García Criado et al. 2025; Keck et al. 2025). Indeed, spatial and temporal biodiversity change among communities can take many forms (Blowes et al. 2024; McGill et al. 2015; Socolar et al. 2016). For instance, biotic homogenisation in a metacommunity under increasing or decreasing levels of biodiversity can be termed additive or subtractive homogenisation for either the gain of the same species or the loss of different species respectively (*sensu* Socolar et al. 2016). Whereas common and widespread species can be linked to additive homogenisation, the loss of rare species can cause subtractive homogenisation (Blowes et al. 2024; Socolar et al. 2016). The extent to which common species appear late in the ecological succession process is further suggested as a driver of biotic homogenisation, emphasising the role of natural, as opposed to anthropogenic, causes of the biodiversity change (Staude et al. 2023). Biodiversity loss can also happen through the process of metacommunity diversification where, for example, the species lost are those in common among the communities (Blowes et al. 2024; Socolar et al. 2016). The many forms that biodiversity change can take within a metacommunity suggest that its interpretation should be done within rigorous theoretically based frameworks.

**FIGURE 1:**
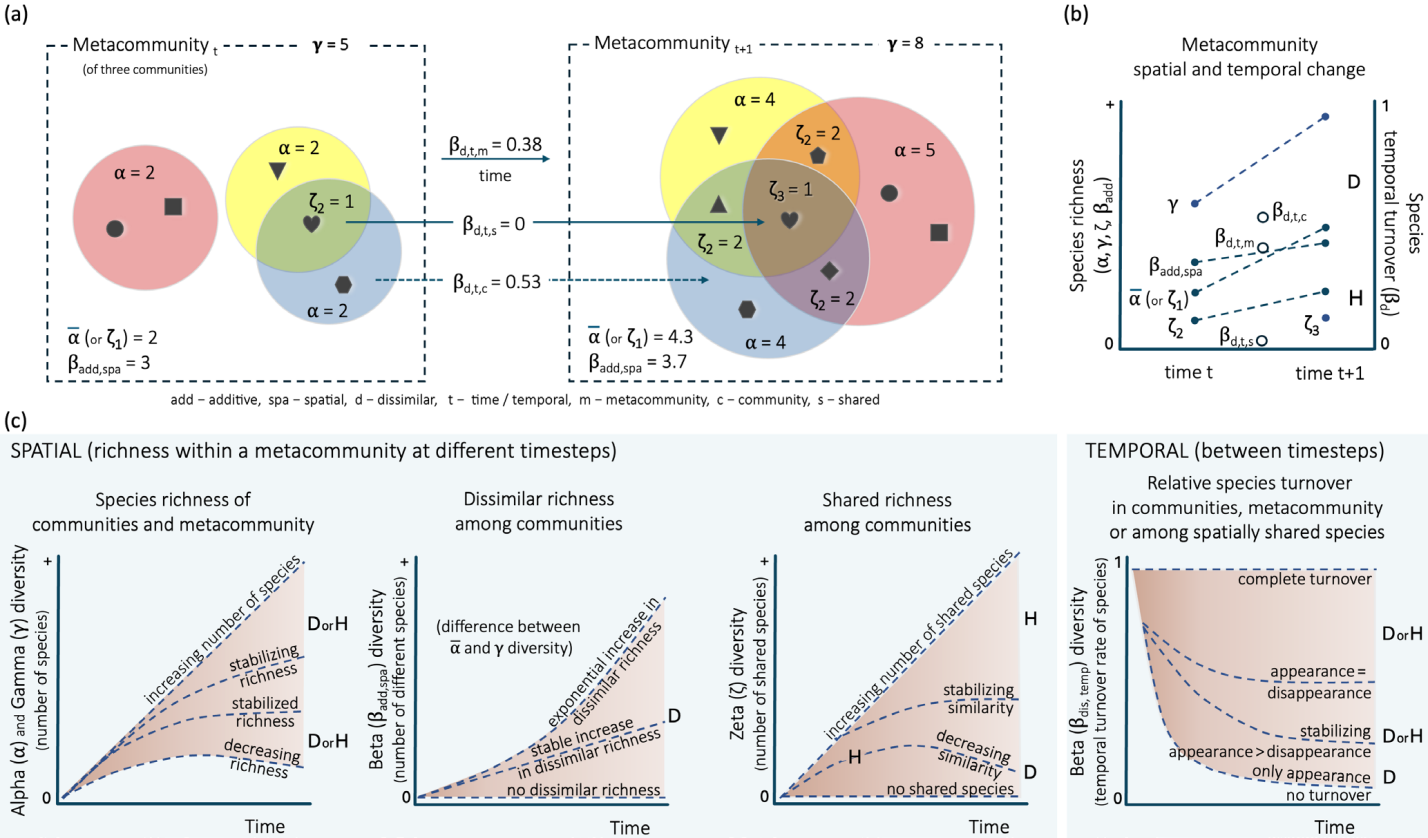
A framework for addressing biodiversity change in a metacommunity. (a) An illustration of the spatio‐temporal partitioning of the biodiversity of a metacommunity at two time‐instances. The spatial components of alpha (𝝰), gamma (𝛄) and zeta (𝛇) diversity are presented at each time‐instance along with additive beta diversity (𝝱_add_) calculated as 𝛄‐ᾱ (Lande 1996). 𝛇 is the average number of species shared among communities (Hui and McGeoch 2014; McGeoch et al. 2019). The temporal component of dissimilar beta diversity (Jaccard dissimilar index), that is, the rate of change in species (or taxa) appearing (immigrating) and disappearing (extirpated or extinct) within communities over time (Hillebrand et al. 2018) (𝝱_d,t_) is presented *between* the two time‐instances. 𝝱_d,t_ is partitioned into the turnover within the metacommunity (𝝱_d,t,m_), in average within all communities (𝝱_d,t,c_) (stapled line in the figure because only one community is exemplified but the values represent the average across all three communities) and within the subset of shared species of the maximum zeta order (𝝱_d,t,s_). (b) A graphical presentation of the illustrated metacommunity. The average community richness (ᾱ), the total species richness (𝛄), the difference in species richness between the communities (𝝱_add_) and the number of shared species in the metacommunity (𝛇) all increase over time, indicating the metacommunity both diversify (D) and homogenise (H), and that the highest temporal species turnover is found at the community level (𝝱_d,t,c_). (c) Ways by which species diversity in a metacommunity may change spatio‐temporally over millennia. The set of biodiversity metrics provide information about metacommunity biodiversity diversification (D) and homogenisation (H), with the coloured fields representing a continuum of possible change—a diversity space. The stapled lines (and areas in between) represent hypothetical metacommunity change at timesteps from the retreat of the ice sheets (see Figure 2) until the present. Change in 𝝰 or 𝛄 over time (left panel). Development of species diversity on average across catchment communities (ᾱ) or their metacommunity (𝛄). Note that this is not equal to the accumulated species diversity, as some taxa may disappear over time. Continuous immigration and hence new species are environmentally filtered through both abiotic conditions and ecological interactions that may cause dynamics in species diversity at both community and metacommunity level (Mittelbach and Schemske 2015). The development of average ᾱ and 𝛄 diversity can therefore take one of several trajectories. Change in spatial 𝝱_add_ over time (second panel) can be derived directly from the 𝛄 and the average ᾱ across the metacommunity as a measure of the difference among the communities. Change in 𝛇 over time (or temporal change in species similarity across communities) (third panel). If, for instance, a set of pioneer species were successful in dispersing to and establishing in all communities at the onset of the Holocene, 𝛇 will equal the set of pioneer species at this time but can never be larger than that of the community with the lowest 𝝰 diversity. At the other extreme, with no shared species among the communities at the onset of the Holocene, and with the species assemblages of all communities remaining unique, 𝛇 would stay at zero. Change in 𝝱_d,t_ over time (last panel). Trajectories in 𝝱_d,t_ are many. It can in principle range from a maximum (𝝱_d,t_ = 1) reflecting a complete turnover of the species assemblage between timesteps, to a steep decline (𝝱_d,t_ > 0) reflecting that all species persist alongside species immigration and a minimum (𝝱_d,t_ = 0) reflecting no turnover. Between the extremes of 0 and 1, 𝝱_d,t_ decline can reflect different processes such as the increase or decrease in the proportion of species with a stable presence over time.

The concepts of alpha, beta and gamma diversity are commonly applied to describe biodiversity change (Wang and Loreau 2014; Whittaker 1972). The alpha and gamma diversity metrics are, in their simplest form, merely counts of taxa at the community and metacommunity level, respectively. Beta diversity metrics provide information about the differences between communities either in time or space (Pinsky et al. 2025) and are contingent on the level of taxonomic identity attained in order to provide information about species turnover (Anderson et al. 2011; Baselga et al. 2007; Koleff et al. 2003; Magurran et al. 2018). Accordingly, spatio‐temporal studies of biotic homogenisation often use measures of beta diversity (Blowes et al. 2024; Daru et al. 2021; Finderup Nielsen et al. 2019; Fraser et al. 2022; Keck et al. 2025; Mori et al. 2018; Yang et al. 2021). For the partitioning of biodiversity among several localities or communities within a metacommunity, zeta diversity has also been applied (Latombe et al. 2017; Simons et al. 2019). Zeta diversity is a metric that captures the number of shared taxa among communities and is flexible in the number of communities that can be compared (Hui and McGeoch 2014). For instance, in a metacommunity of three communities, the spatial beta diversity (measured as additive beta diversity, that is, the difference between gamma diversity and the average alpha diversity) and zeta diversity (either measured at order two, that is, the average number of species shared between two communities and equal to Jaccard's similarity index, a beta diversity index, or at the order three, that is, the number of species shared among all three communities and unique to zeta diversity) can provide complementary information. For instance, an increase in both would suggest both diversification and homogenisation (Figure 1a,b). Hence, to disentangle more subtle changes such as additive homogenisation (Figure 1a,b) or other forms of spatio‐temporal change (Figure S1), we propose the inclusion of zeta diversity within a framework that typically consists of alpha, beta and gamma diversity.

Temporal biodiversity change is, however, challenging to quantify, especially over long timescales. A lack of data, in particular from the past, causes a lack of biodiversity baselines towards which current and future biodiversity can be evaluated (Fordham et al. 2020; Magurran et al. 2010, 2019). As temporal changes in biodiversity are interlinked with spatial patterns, care should be used when substituting space for time (Damgaard 2019). Accordingly, the depiction of biodiversity change and novelty depends on both spatial and temporal baselines (Radeloff et al. 2015). Long‐term adaptive monitoring, which can provide such biodiversity baselines, is rare (Ims and Yoccoz 2017; Lindenmayer and Likens 2009) and data that provide such baselines from the past need to be interpreted with caution to avoid biased inferences of biodiversity change (Kapfer et al. 2017). Empirical studies that permit assessment of biodiversity change are hence very valuable, with paleoecological studies providing unprecedented ecological insights.

Pollen‐based paleoecological studies have provided information about shifting baselines of biodiversity (Allen and Huntley 1999; Birks and Birks 2000; Birks et al. 2016; Felde et al. 2018; Giesecke et al. 2019; Seppä et al. 2009; Willis et al. 2010). However, pollen‐based studies are limited and biased by low taxonomic resolution (typically to the family or genus) and long‐distance dispersed pollen, limiting single community assessments due to the non‐independence of site data. Samples can also be swamped by high pollen‐producing taxa such as trees and grasses, and under‐represent low pollen production and insect‐pollinated species (Birks et al. 2016; Felde et al. 2015; Reitalu et al. 2019). In comparison, plant sedimentary ancient DNA (*sed*aDNA) has a more local and spatially delimitable source area, fewer problems with swamping, and an average higher taxonomic resolution often to species. Species richness values estimated using sedaDNA from recently deposited sediments have been found to correlate with that of modern local vegetation (Alsos et al. 2018; Sønstebø et al. 2010). When the two approaches are compared, *sed*aDNA better reflects the local plant community and detects more herbaceous species and more taxa overall than pollen analyses (Clarke et al. 2019; Sjögren et al. 2017; Zimmermann et al. 2017a, 2017b). Thus, *sed*aDNA provides an improved basis from which we can detect spatio‐temporal biodiversity changes in metacommunities of the past.

In this study, we explore how the set of alpha, beta, gamma and zeta diversity metrics behave in a plant metacommunity spanning the last 13 millennia in northern Fennoscandia. This time period covers the retreat of the glacial ice that had covered nearly all of Fennoscandia to the present. We particularly focus on the metacommunity processes of diversification and homogenisation (Figure 1c), using *sed*aDNA data from lake sediments that have been identified using reference libraries constructed from the extant Nordic flora. Because each lake has accumulated *sed*aDNA from its catchment, we define a community as the species assemblage from a lake catchment and define the metacommunity as the full set of lake catchments. Since the ice retreat, regional increases in both the number of taxa (Rijal et al. 2021) and trait diversity during the Early Holocene (Alsos et al. 2022) have already been documented. Here, we ask to what extent the metacommunity biodiversity has been diversifying or homogenising from the time of ice retreat and primary succession towards the present‐day ecosystem (Figure 1c and Figure S1). Because the geographical distances among the communities vary, we also ask to what extent there is distance decay in biodiversity (Hui and McGeoch 2014; McGeoch et al. 2019), comparing time and distance to the changes in biodiversity within the metacommunity.

## 2 Materials and Methods

### 2.1 Study Area, Current Vegetation and Time Span of Lake Sediment Records

**FIGURE 2:**
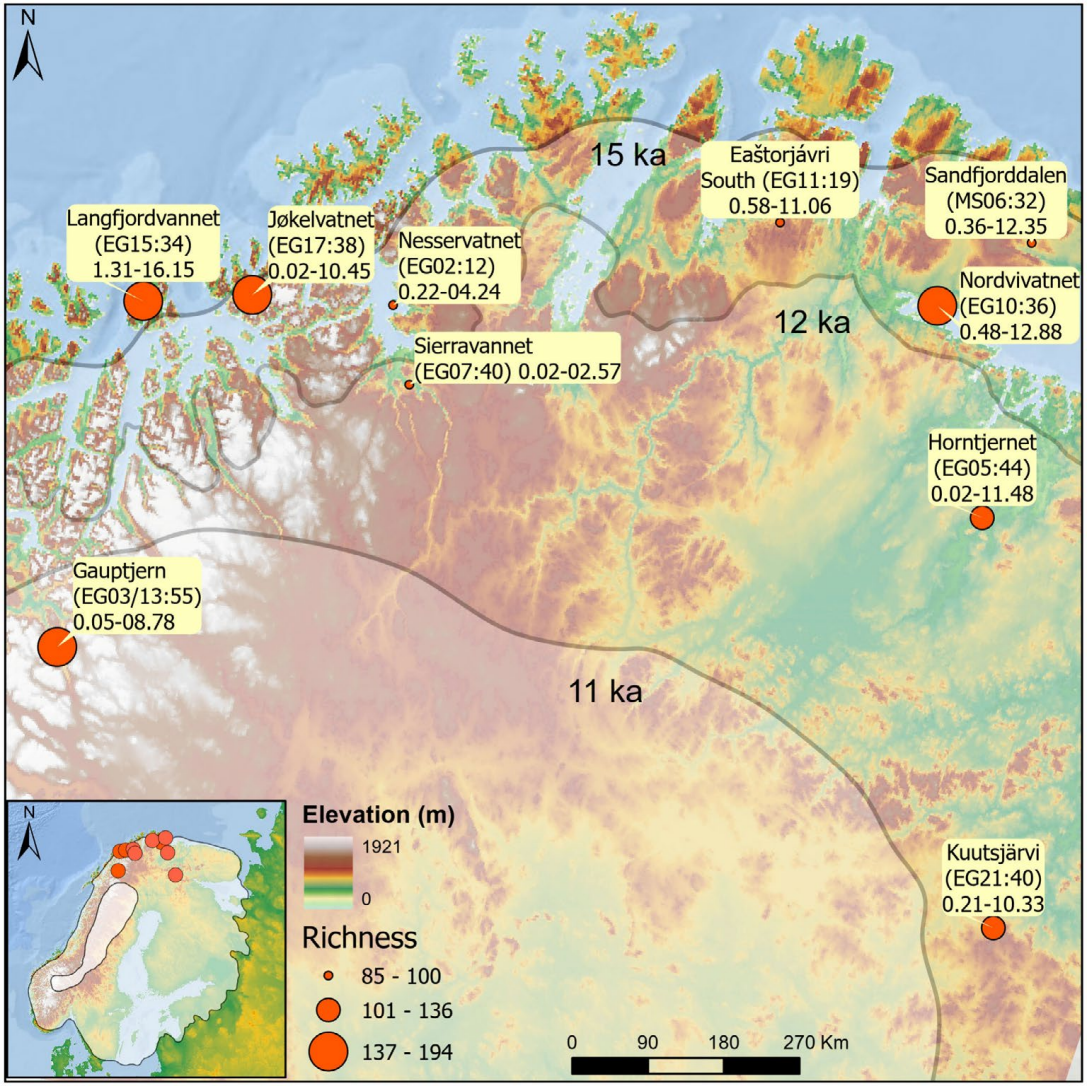
Digital elevation map of the study area [data source: European Environment Agency (EEA)]. The most credible generalised extent of the Scandinavian ice sheet (Hughes et al. 2016) at 15, 12 and 11 ka are indicated by transparent layers. Lake locations are depicted with points scaled by the total taxa found in the lake. Lakes are further depicted with their names, codes and number of samples within the parentheses, and the temporal span of each sediment core in thousands of years before present (ka). Inset shows the extent of the Scandinavian ice sheet at 15 and 10 ka. See Table S1 for lake metadata.

The study area covers northernmost Fennoscandia above the Arctic Circle (67.75–70.43 N, 19.62–30.02 E) (Figure 2). The sediment cores from the 10 study lakes (nine in Norway and one in Finland) vary in their age range, with the oldest starting at 16.1 thousand calibrated years before present (ka), but most covering the majority of the Holocene (starting at 11.7 ka) (Table S1). The current catchment vegetation of lakes Sandfjorddalen, Eaštorjávri South, Langfjordvannet and Jøkelvatnet is dominated by treeless heath, shrub, meadow and mire. Gauptjern, Nesservatnet and Nordvivatnet are surrounded by birch ( *Betula pubescens* Ehrh.) forests, but also heath, meadow and mire. Horntjernet, Sierravannet and Kuutsjärvi are surrounded by mixed boreal forests, heath and mire, with pine ( *Pinus sylvestris* L.; all three lakes) and spruce ( *Picea abies* (L.) H. Karst.; latter two lakes) present. The 10 lake sites are located in the present‐day ecotone of northern Boreal birch‐conifer vegetation and the open‐shrub tundra and are defined as belonging to the ecoregion of Scandinavian Montane Birch forest and grasslands (https://ecoregions.appspot.com, Dinerstein et al. 2017).

### 2.2 The *sed*aDNA Dataset

We analysed a previously published terrestrial plant *sed*aDNA dataset originating from sediment cores of the 10 lakes. Out of the 355 *sed*aDNA samples used in this study, 316 were published by Rijal et al. (2021) and the remaining 39 by Alsos et al. (2022). The taxonomic assignment of some *sed*aDNA sequences published in Rijal et al. (2021) was revised by Alsos et al. (2022) using the PhyloNorway DNA reference library for vascular plants (Alsos et al. 2020). Altogether, the terrestrial plant dataset consists of 213 vascular plants and 77 bryophytes. We converted semi‐quantitative PCR replicate data of the 290 terrestrial plant taxa originating from 350 distinct sediment samples (five DNA extraction duplicates were excluded) into presence/absence data to represent the plant communities analysed in this study (Table S1).

Taxon detection in *sed*aDNA analysis was expected to be biased by sample age due to increased DNA degradation with time (Capo et al. 2021). Both DNA degradation and PCR biases can therefore directly affect the estimation of our diversity metrics. However, the data used here were based on eight PCR replicates to maximise taxon detection (Alsos et al. 2022; Rijal et al. 2021). We highlight that there was minimal to no age bias effect on the samples, and detection bias was minimised by applying quality control metrics to filter out poor quality samples (see Rijal et al. 2021). Finally, the entire dataset was harmonised using standardised taxonomy (Alsos et al. 2022; Rijal et al. 2021), assuring a reliable database for estimating diversity metrics within and among the lake sediment records.

### 2.3 Calculation of Biodiversity Metrics

The total number of unique taxa from each sediment sample was used to represent a community in a window of time. A metacommunity approach was taken by using the sediment samples from each of 8 lakes (for the Early and Middle Holocene; two records did not cover this time interval) and 10 lakes (for the Late Holocene), where the set of all lakes represented a metacommunity. To capture biodiversity change over time at the metacommunity level, the total number of unique taxa from each sediment sample were binned into time intervals for the total set of lakes. Bins were made for each 500‐year time interval for all analyses, except for the distance‐decay analysis where bins were made for each of the three climate‐related Holocene subdivisions, that is, each 4000‐year time interval from 11.7 ka to present (Walker et al. 2012).

Alpha diversity was calculated as the number of taxa per sediment sample in each community (Rijal et al. 2021), and then averaged across all lakes within each time bin to have an estimate of average alpha diversity for each 500‐year interval throughout the Holocene. Gamma diversity was calculated as the total number of taxa at the metacommunity level (at each 500‐year interval and with one estimate per timestep only). Whittaker's additive beta diversity or spatial beta diversity, was calculated for each 500‐year interval following Lande (1996).

Temporal beta diversity (species turnover) was calculated as Jaccard's dissimilarity, or the species exchange ratio, between each 500‐year interval within each community, with β_dis_ = (*S*_imm_ + *S*_ext_)/*S*_tot_ (Hillebrand et al. 2018; Koleff et al. 2003), where *S*_imm_ is immigration or taxa appearing between time‐consecutive samples, *S*_ext_ is extinction or taxa disappearing between time‐consecutive samples, and *S*_tot_ is the total number of detected taxa of two time‐consecutive samples. We calculated temporal beta diversity across the timesteps between consecutive 500‐year intervals as the average per community, as the total for the entire metacommunity, and across the subset of shared taxa within the metacommunity (Figure 1a,b).

Zeta diversity was calculated as the number of taxa shared among the communities (Hui and McGeoch 2014; McGeoch et al. 2019) at each 500‐year interval, comparing two, three and more catchment communities until all taxa of all communities at each interval were compared. Note that for zeta diversity estimates of the total metacommunity, that is, with all communities compared, there was only one estimate of zeta diversity per interval possible. The number of communities compared is termed the zeta order. Because a zeta order of 1 equals the average alpha diversity among communities, it was omitted. The maximum zeta order applied increases from 7 to 8 to 9 from Early to Middle to Late Holocene, respectively.

We also included an analysis of the zeta ratio, calculated as the ratio of zeta diversity of order *n* + 1 divided by zeta diversity of order *n* (Hui and McGeoch 2014; McGeoch et al. 2019) at each 500‐year time interval. The zeta ratio therefore provides information as to the species turnover within the subset of shared species at any time instance (Latombe et al. 2017). This is also considered as a retention rate across the metacommunity of rare to common species (McGeoch et al. 2019).

Finally, we assessed distance decay, that is, how zeta diversity was affected by the Euclidean distance among different communities, for the same set of samples over the three Holocene subdivisions, thereby excluding Late Glacial samples (> 11.7 ka) that were only present at three lakes (Table S1).

### 2.4 Statistical Analysis

To test whether the diversity analyses were impacted by the increasing number of communities over time, largely related to the glaciation history (Figure 2), we created subsets of data by randomly subsampling from the full dataset without replacement. However, as there were few communities during the Late Glacial time interval, we generated a set of six datasets based on resampling of 1–5 communities, and with resampling starting at the time interval the metacommunity had sufficient communities for resampling (see Figure S2 for subsampling details). The gamma, beta and zeta diversities were calculated from each dataset using 100 subsampling iterations. The average values from these repetitions were then compared to those derived from the full dataset (Figures S2 and S3). Similar trends in gamma and zeta diversities, as well as comparable temporal beta diversity patterns, were observed regardless of the number of communities included (Figures S2 and S3). Therefore, we present results based on the raw data with a minimum of two communities per 500‐year intervals, but we also include data adjusted for the increasing number of communities over time to ensure robustness and transparency in our analyses.

The temporal trends of all diversity metrics were modelled using generalised additive models (GAMs). For alpha and beta diversity (for each community), sample‐wise estimates were used as the response variable and the age of the sample as the predictor variable. For gamma diversity, and for zeta diversity and ratio (orders 2–8), we used 500‐year interval estimates at the metacommunity level as the response variable and the age of the time intervals as the predictor variable. The mean alpha, sampling adjusted gamma and zeta diversity estimates were rounded to the nearest whole numbers prior to modelling to retain properties of counts (e.g., increasing variance with the mean). For GAMs of zeta diversity and the zeta ratio, the zeta order of 9 was excluded as there were too few time intervals to fit the GAM. We initially used the Poisson distribution for modelling alpha, gamma and zeta diversities. However, due to overdispersion, we changed the distribution to either a negative binomial or Tweedie distribution in order to improve model fit (Figure S4). Excluding the oldest sample and using log transformed taxonomic richness as the response variable in a linear model improved the model fit in Nesservannet. A Poisson GAM for gamma diversity and a Tweedie GAM for the mean alpha diversity improved model fit (Figure S5).

When calculating temporal beta diversity for the subset of shared taxa, we applied the list of taxa as defined by the zeta diversity of the total metacommunity per 500‐year interval. Note that the maximum number of communities, or the maximum zeta order with shared taxa, could vary between consecutive 500‐year intervals (see Figure 1a for an illustration).

A binomial distribution was primarily used while modelling temporal beta diversity, that is, the proportional ratio of counts (see Douma and Weedon 2019). Removal of the most recent sample of Kuutsjärvi and application of a Gaussian distribution to both Kuutsjärvi and Nesservannet improved the model fit at catchments while analysing the temporal beta diversity pattern through time (Figure S6). A Gaussian GAM for temporal beta diversity at the metacommunity level, and a linear model for beta diversity of the shared taxa as well as for the mean temporal beta diversity at 500‐year intervals provided better fits than a binomial distribution (Figure S7). A Tweedie GAM was needed to improve the model fit in one of the analyses of the temporal pattern of zeta diversity (Figure S8) and GAMs with beta regression provided a reasonable model fit (Figure S9) while analysing the temporal pattern of zeta‐ratio (see Douma and Weedon 2019). We performed all analyses in R (R Core Team 2023): the GAM analyses used the *mgcv* package (Wood 2017), the diagnostic plots for all the regression models were generated using *DHARMa* (Hartig 2022), and the temporal trends of all diversity metrics were visualised using *ggplot2* (Wickham 2016).

## 3 Results

**FIGURE 3:**
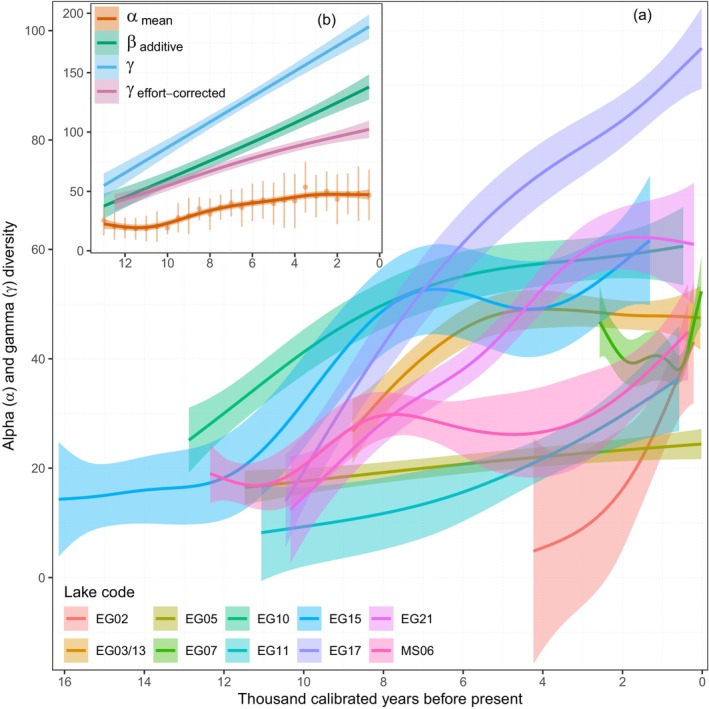
Alpha, beta and gamma diversity during the Holocene. (a) Taxonomic richness of terrestrial plants (alpha diversity) in single communities over time, and (b) the average alpha (±1 SD), beta (Whittaker's additive beta diversity), gamma, and sampling adjusted gamma diversity across the metacommunity over time. The metacommunity estimates are based on sediment samples binned at 500‐year intervals from minimum two lakes. The sampling adjusted gamma was estimated using three lakes per 500‐year interval for the last 12.5 ka after 100 subsampling iterations. Shadings indicate 95% confidence intervals of the fitted models. See Figure S10 for raw data points and Figure 2 for lake names.

The size of the metacommunity, in terms of the number of constituent communities, increased over time as the glacial ice retreated and the sampling sites could be colonised, although two records begin in the Late Holocene (Figure 2). Still, as also shown by Rijal et al. (2021), the different communities had different rates of alpha diversity (number of taxa) change over time independent of when their records began, with an increase in all communities except Sandfjordalen and Langfjordvatnet during the Middle Holocene and Gauptjern, which stabilised in the Late Holocene towards the present (Figures 1 and 3a and Table S2). At the metacommunity level, the average alpha diversity had a steady increase throughout the Holocene (*F* = 57.0, effective df (edf) = 6.44, *R* ^2^_adj_ = 0.93, *p* < 0.0001, Figure 3b and Figure S10b). The total number of taxa at the metacommunity level (gamma diversity) also increased (χ^2^ = 336.9, edf = 5.29, *R* ^2^_adj_ = 0.92, *p* < 0.0001) at a rate far exceeding that of the average alpha diversity. Metacommunity size adjusted gamma diversity (based on three communities) also increased over time, though at a lower magnitude than gamma diversity (χ^2^ = 119.2, edf = 2.42, *R* ^2^_adj_ = 0.88, *p* < 0.0001, Figure 3b). Consequently, the additive beta diversity increased throughout the Holocene (*F*_1,24_ = 147.6, *R* ^2^_adj_ = 0.85, *p* < 0.0001, Figure 1a,b and Figure S11), as the distance between the average alpha and the gamma diversity increased over time (Figure 3b).

**FIGURE 4:**
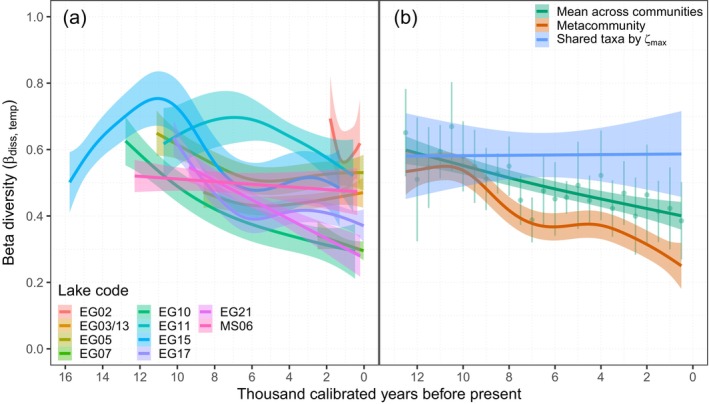
Temporal beta diversity during the Holocene. The terrestrial plant taxa turnover (Jaccard's dissimilarity) between successive samples over time in (a) each of 10 communities, and (b) across the metacommunity including mean temporal beta diversity across local communities and the temporal beta diversity among the subset of shared taxa in the metacommunity. The subset of shared taxa applied here are those shared among the maximum number of communities at each time interval. The metacommunity estimates are based on sediment samples binned at 500‐year intervals from minimum two lakes. Shadings indicate 95% confidence intervals of the fitted models. See Figure S11 for raw data points and Figure 2 for lake names.

The taxon exchange over time, the temporal beta diversity, changed both within and among the communities (Figure 4a and Figure S12a), with an increasing trend in the Late Glacial period in Langfjordvannet and with most communities showing a significant decline during the Holocene (Table S3). At the metacommunity level, the taxon exchange between time instances showed a clear decline throughout the Holocene (Figure 4b and Figure S12b), both when calculated as the average taxon exchange across communities (*F*_1,23_ = 37.46, *R* ^2^_adj_ = 0.60, *p* < 0.0001) and at the metacommunity level (*F* = 15.17, edf = 5.70, *R* ^2^_adj_ = 0.81, *p* < 0.0001). Trends in both plant taxon appearance (immigration) and disappearance (local extinction) in the metacommunity increased over time (Figures S13 and S14), with plant taxon appearance being slightly higher than disappearance and hence causing the overall decline in temporal turnover.

**FIGURE 5:**
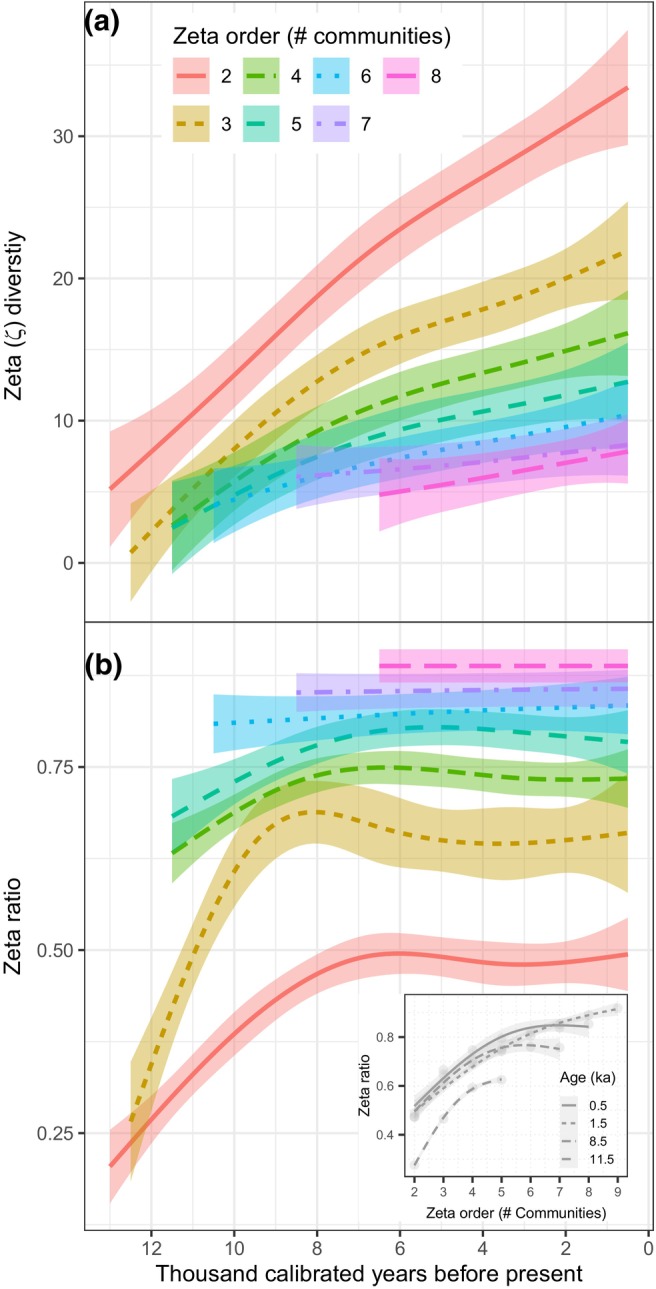
Zeta diversity and zeta ratio during the Holocene. (a) Zeta diversity over time for 10 communities. Each line shows the average number of shared terrestrial plant taxa among two (zeta order of 2) and up to eight (zeta order of 8) communities. (b) Zeta ratio over time for 10 communities. Each line shows the proportion of taxa that are shared between two neighbouring zeta orders. The inset shows the retention rate, the relation between zeta ratio, and zeta order, at four distinct time‐intervals. All estimates are based on sediment samples each binned at 500‐year intervals. Shadings indicate 95% confidence intervals of the fitted generalised additive models. See Figure S14 for raw data points.

The number of shared taxa among communities, the zeta diversity, increased with time throughout the Holocene (Figure 5a). The highest rate of increase was found for the average of any two communities, that is, the zeta order of 2, whereas the rate levelled off markedly as more communities were compared, that is, as the zeta order increased (Figure 5a and Figure S15a). The subtle reduction in the rate of increase in zeta diversity for higher orders was further indicated by their non‐significant smoothing terms (Table S4).

The rate at which the taxa shared among communities was retained at the metacommunity level, the zeta ratio, shifted with time only in the Early Holocene (inset Figure 5b, Figure S15b and Table S5), suggesting a shift from rare to more common taxa being shared. The retention rate in the Middle and Late Holocene was higher and did not change over time (Figure 5b), suggesting the balance between rare and common taxa being shared among communities stabilised.

Temporal beta diversity was also calculated for the subset of shared species in the metacommunity (Figure 1). The exchange rate between time instances was high within this subset of taxa and did not change throughout the Holocene (*F*_1,23_ = 0.00, *R* ^2^_adj_ = −0.04, *p* > 0.05, Figure 4b). Only a few taxa were consistently shared among the communities through time (Table S6).

**FIGURE 6:**
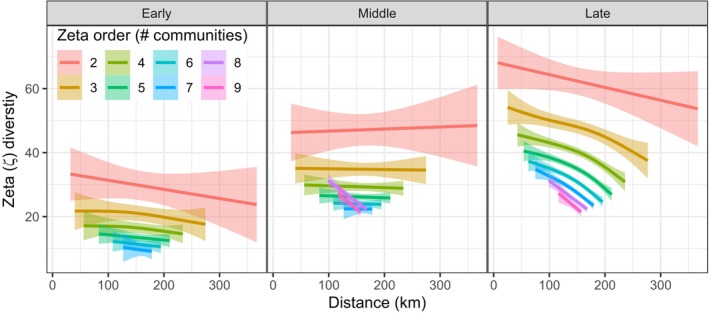
Zeta diversity with distance during the Early, Middle and Late Holocene. Each line shows the average number of shared terrestrial plant taxa in relation to distance between communities, among two (zeta order of 2) and up to nine (zeta order of 9) communities. Shadings indicate 95% confidence intervals of the fitted generalised additive models. See Figure S15 for raw data points.

Zeta diversity was related to distance between communities (Figure 6), but this is only clear in the Late Holocene as shown by the decreasing number of taxa being shared with increasing distance among communities (Figure 6 and Figure S16) and statistically significant smooth terms (Table S7).

## 4 Discussion

We applied a set of complementary diversity metrics to a unique spatio‐temporal dataset of plant *sed*aDNA taxa and found the complementarity provides novel insights into the plant diversity changes that took place in northern Fennoscandia throughout the Holocene. First, we find that the number of taxa at the community level (average alpha), at the metacommunity level (gamma), the difference between communities (additive, spatial beta), and taxa shared among the communities (zeta) all increased continuously from the time of the ice retreat to the Late Holocene. Hence, the metacommunity diversified over millennia, both in overall taxon richness and in taxon difference between its communities. Concurrently, the metacommunity homogenised. Both processes were additive in the sense that both the diversification and the homogenisation happened under increasing taxon richness in the metacommunity (Socolar et al. 2016). The homogenisation decreased as more communities were compared (with increasing zeta order), and so we can conclude that homogenisation differed within the metacommunity. However, these patterns are based on mere counts of taxa at each 500‐year interval since the ice retreat. When also addressing taxon turnover between time intervals or temporal dissimilarity, we found a growing proportion of the taxa was persistent over time at the community and metacommunity levels. Yet, among the subset of shared taxa in the metacommunity, the turnover was continuously high throughout the Holocene. Our findings thus suggest that a metacommunity can remain spatially diverse over millennia despite that biotic homogenisation takes place among its communities. Furthermore, our findings suggest that not all communities in a metacommunity are equally involved in the homogenisation process and that taxa being involved in biotic homogenisation at one time instance does not necessarily imply that they are persistent in the homogenisation over time.

The plant diversity changes of the metacommunity are clearly linked to the development of the communities following the ice retreat of the last glaciation (Alsos et al. 2022; Birks et al. 2012; Huntley et al. 2013), with the taxon richness at the community level (alpha diversity) starting close to zero and increasing. These mass effects (*sensu* Leibold et al. 2004) could result from a growing regional species pool enabling species to establish in environmentally sub‐optimal habitats, thereby elevating local richness. As the Holocene proceeded, taxon richness at the metacommunity level (gamma diversity) increased manyfold and more than the average taxon richness per community. This discrepancy indicates a large increase in the number of taxa being different between the communities (spatial beta diversity) and shows the metacommunity consistently diversified throughout the Holocene. This further suggests that the development of the metacommunity over time was consistent with spatial niche separation through priority effects (Chase 2003), including niche construction (Fukami 2015; Odling‐Smee et al. 2013) in which biota are directing the community change, and/or species sorting among its communities, in which environmental differences cause communities to differ (Leibold et al. 2004; Mori et al. 2018). The increase in taxon richness at both the community and metacommunity levels continued throughout the Holocene, suggesting the species pool at both the local and the regional scale may still not be saturated (Alsos et al. 2022; Rijal et al. 2021), further suggesting the diversity changes of this northern Fennoscandian metacommunity have been impacted by the addition of new taxa over millennia.

Biotic homogenisation during the Holocene has previously been found among assemblages of North American mammalian faunas, with two distinct phases in which the first followed the extinction of megafauna and the second the rise of human impact (Fraser et al. 2022). In contrast, the plant metacommunity homogenisation observed in our study happened in a region with low human population size and growth (Brown et al. 2022). Furthermore, the number of shared taxa increased alongside metacommunity richness, which can therefore be classified as additive biotic homogenisation in which the communities become more homogenous through the addition of new shared species (Socolar et al. 2016). However, less than 10 taxa were shared among all the communities in any 500‐year time window, a fraction of the average taxon richness (average alpha) in the metacommunity. The biotic homogenisation may thus not have caused a decrease in the multi‐functionality of the metacommunity (*sensu* Mori et al. 2018), as the set of functions provided with the shared taxa were in addition to a growing set of unique taxa in the communities (Alsos et al. 2022). Furthermore, although the biotic homogenisation was linked to primary succession (emphasising a natural way by which homogenisation can take place; Staude et al. 2023), the continued differentiation of taxa among communities within the metacommunity suggests succession was limited as a homogenisation force. Importantly, we found a high turnover between time intervals among the shared taxa. Hence, the set of shared taxa shifted, especially among forbs, whereas shared woody taxa that would be linked to late successional stages showed a higher consistency over time (see Table S6).

The biotic homogenisation, as identified by the zeta‐diversity of order two, that is, a pairwise comparison of communities, is equal to a beta‐metric providing pairwise similarity of community assemblages (McGeoch et al. 2019). Hence, a beta diversity metric such as Jaccard's similarity (Koleff et al. 2003), not included in our study, would suffice to capture homogenisation. However, zeta diversity of higher orders provided more insight as to how the biotic homogenisation was distributed among communities. For instance, there was no significant increase in biotic homogenisation over time when all communities were compared, whereas the average of subsets of up to five communities did show a significant increase. This suggests that biotic homogenisation was stronger among a subset of the metacommunity. The number of shared taxa (zeta) declining with distance, that is, the distance decay between communities, in the Late Holocene, could be contributing to this spatio‐temporal pattern in biotic homogenisation. Indeed, distance between communities can affect patterns of movement within a metacommunity (*sensu* MacArthur and Wilson 1967; Storch and Okie 2019), for example, through dispersal distance, low connectivity or environmental distance (Chase 2003). In contrast, the retention rate (zeta‐ratio) provided little additional spatio‐temporal information in our study. The zeta diversity already showed it was only a subset of the shared taxa that were shared among all communities and hence widespread (Blowes et al. 2024). The zeta‐diversity of higher orders clearly provided unique information about biotic homogenisation.

The decreasing turnover between successive assemblages indicates an increasing proportion of the taxa remained in the metacommunity as the Holocene developed. The finding that more taxa appeared than disappeared over time further suggests that the proportion of taxa co‐existing in the local communities also increased over time. Biodiversity per se can be a stabilising force promoting species coexistence (Loreau et al. 2022) and ecosystem functioning, as argued in the insurance hypothesis (Yachi and Loreau 1999). The number of taxa does, however, not necessarily imply stability of ecosystem functioning, as this depends on the traits present (Chao and Colwell 2022; Mori et al. 2018). For the metacommunity of this study, plant diversity of major trait groups showed no change after 9 ka (Alsos et al. 2022) whereas biotic homogenisation continued; hence we can speculate that homogenisation did not contribute to the addition of new, shared functionality among the communities. Rather, the constant addition of new plant taxa, some of which were shared among the communities, suggests that niche complementarity has been ongoing for millennia and possibly enhanced by a range of ecological interactions as the communities have diversified.

Studies from more recent deglaciation events have found positive links between plant communities, abiotic conditions and the biodiversity of other kingdoms (Ficetola et al. 2024). This underscores the importance of plants as primary producers, and so we consider the changing biodiversity documented here as being informative at the ecosystem level. However, it is important to note that in this study any fluctuations in biodiversity at decadal timescales are smoothed. Hence, studies examining changing biodiversity over decadal timescales from more recent deglaciation events (Cantera et al. 2024) or from changing vegetation compositions in northern Fennoscandia (Bråthen et al. 2024) are considered not directly comparable to the results of this study. Here we rather emphasise the role of complementary biodiversity metrics in capturing spatio‐temporal change as an important insight.

In conclusion, plant biodiversity changes in northern Fennoscandia during the Holocene are informative for biodiversity changes in our time. First, communities can remain unique for millennia. Hence, the loss of habitat in one community indicates loss of biodiversity to the metacommunity, which strengthens the argument that habitat loss through human influence causes ecosystem decay and biodiversity loss (Chase et al. 2020). From a practical perspective, the fact that communities can remain unique in their assemblages of taxa through millennia highlights the risk of extrapolating information from one community to another (McGill 2019). Second, novel ecosystems can continuously develop over millennia (Burke et al. 2019), as is the case for our metacommunity as interpreted from diversification throughout the Holocene. Finally, our findings emphasise that additive homogenisation and additive diversification are not mutually exclusive processes in a metacommunity, highlighting the need for complementary metrics for analysing diversity change.

## Author Contributions

I.G.A., K.A.B. and N.G.Y. raised the funding. K.A.B., D.P.R., I.G.A., A.G.B., N.G.Y. and P.D.H. designed the research. K.A.B. conceptualised the analysis. D.P.R. did the statistical analyses. D.P.R. drafted the methods, statistical analysis and results sections and K.A.B. drafted the introduction and discussion sections of the manuscript. All authors have reviewed and approved the final manuscript.

## Conflicts of Interest

The authors declare no conflicts of interest.

## Peer Review

The peer review history for this article is available at https://www.webofscience.com/api/gateway/wos/peer‐review/10.1111/ele.70218.

## Supporting information


**Data S1:** ele70218‐sup‐0001‐DataS1.pdf.

**Table S1:** ele70218‐sup‐0002‐TableS1.xlsx.

**Table S2:** ele70218‐sup‐0003‐Table_S2‐S7.xlsx.

## Data Availability

All the data used in this study are archived as the Dryad dataset and can be accessed from https://doi.org/10.5061/dryad.d51c5b0d6.
