# Supplementary material for: Millennia of Metacommunity Diversification and Homogenization Captured by Sedimentary Ancient DNA
Source: Ecol Lett. 2025 Sep 22;28(9):e70218. doi: 10.1111/ele.70218 (PMC12453138; doi:10.1111/ele.70218)
Supplement: Supplementary file 1 — Data S1: ele70218‐sup‐0001‐DataS1.pdf. [file ELE-28-0-s001.pdf]

Millennia of metacommunity diversification and homogenization captured by sedimentary ancient DNA

Dilli P. Rijal[1,$], Antony G. Brown[1], Nigel G. Yoccoz[2], Peter D. Heintzman[1,3,4], Inger G. Alsos[1], Kari Anne Bråthen[2,$]

Affiliations

[1]The Arctic University Museum of Norway, UiT-The Arctic University of Norway, Tromsø, Norway
[2]Department of Arctic and Marine Biology, UiT-The Arctic University of Norway, Tromsø, Norway
[3]Centre for Palaeogenetics, Svante Arrhenius väg 20C, SE-10691 Stockholm, Sweden
[4]Department of Geological Sciences, Stockholm University, SE-10691, Stockholm, Sweden

[$]These authors contributed equally to the manuscript.

Supplementary materials

Supplementary figures

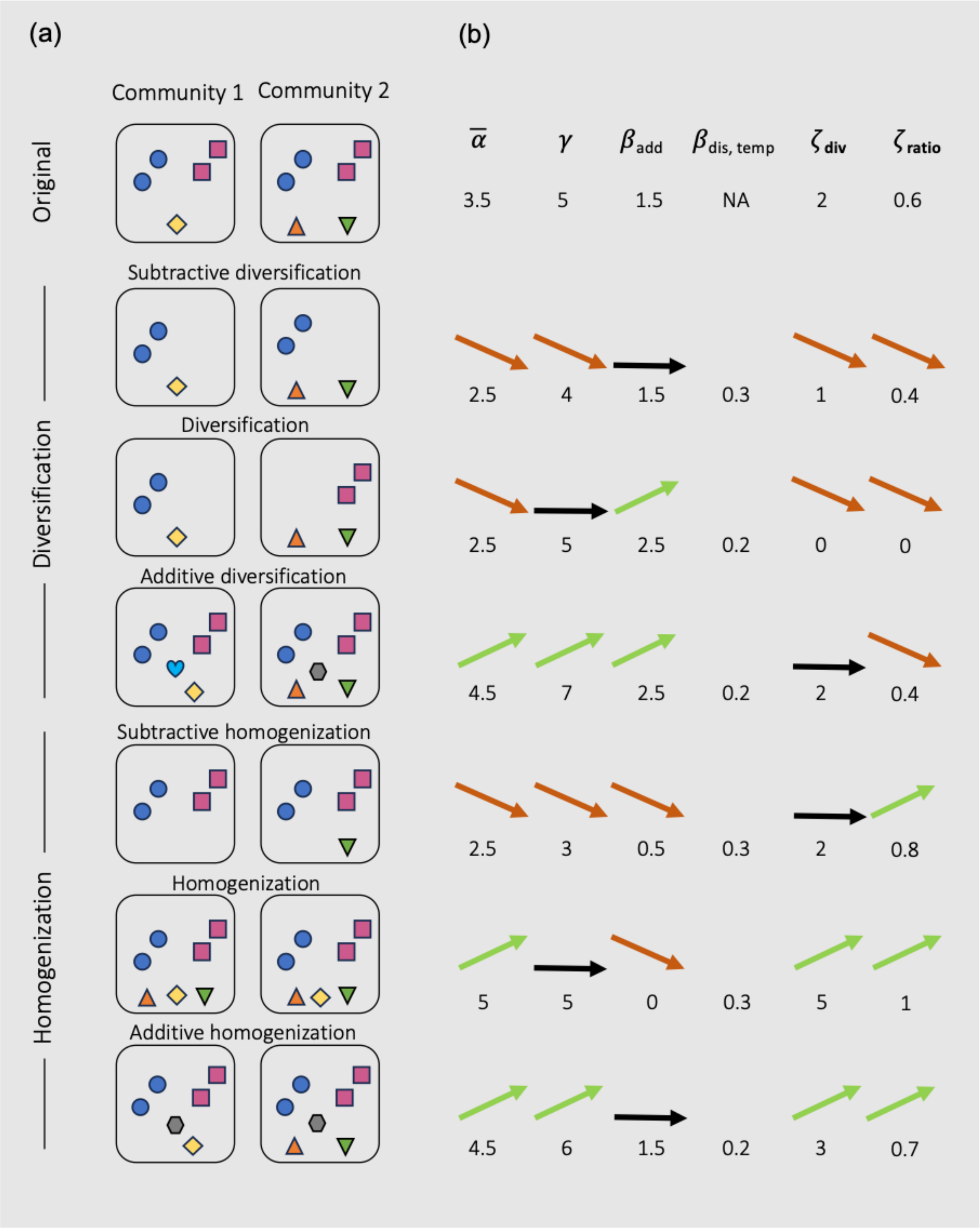

Fig. S1 Biodiversity change in a metacommunity of two communities described by a set of biodiversity metrics. (a) An original metacommunity, or a reference baseline, and how it may change. Each square with colorfilled shapes symbolises different species within a community. The combined changes in the two communities from an original (reference) baseline to a later state are depicted as either differentiation or homogenization (modified from Socolar *et al.* 2016). (b) Average alfa-,

gamma-, spatial and temporal beta-, zeta-diversity and zeta-ratio are calculated for each of six types of metacommunity change, and with arrows showing whether the change caused an increase (green arrow), decrease (red arrow), or no effect (black arrow) on the diversity metrics. Spatial beta diversity is the Whittaker's beta diversity as modified by Lande (1996), the difference between alpha- and gamma diversity, whereas temporal beta diversity is calculated as the Jaccard dissimilarity (Koleff *et al.* 2003), also termed species exchange ratio (Hillebrand *et al.* 2018).

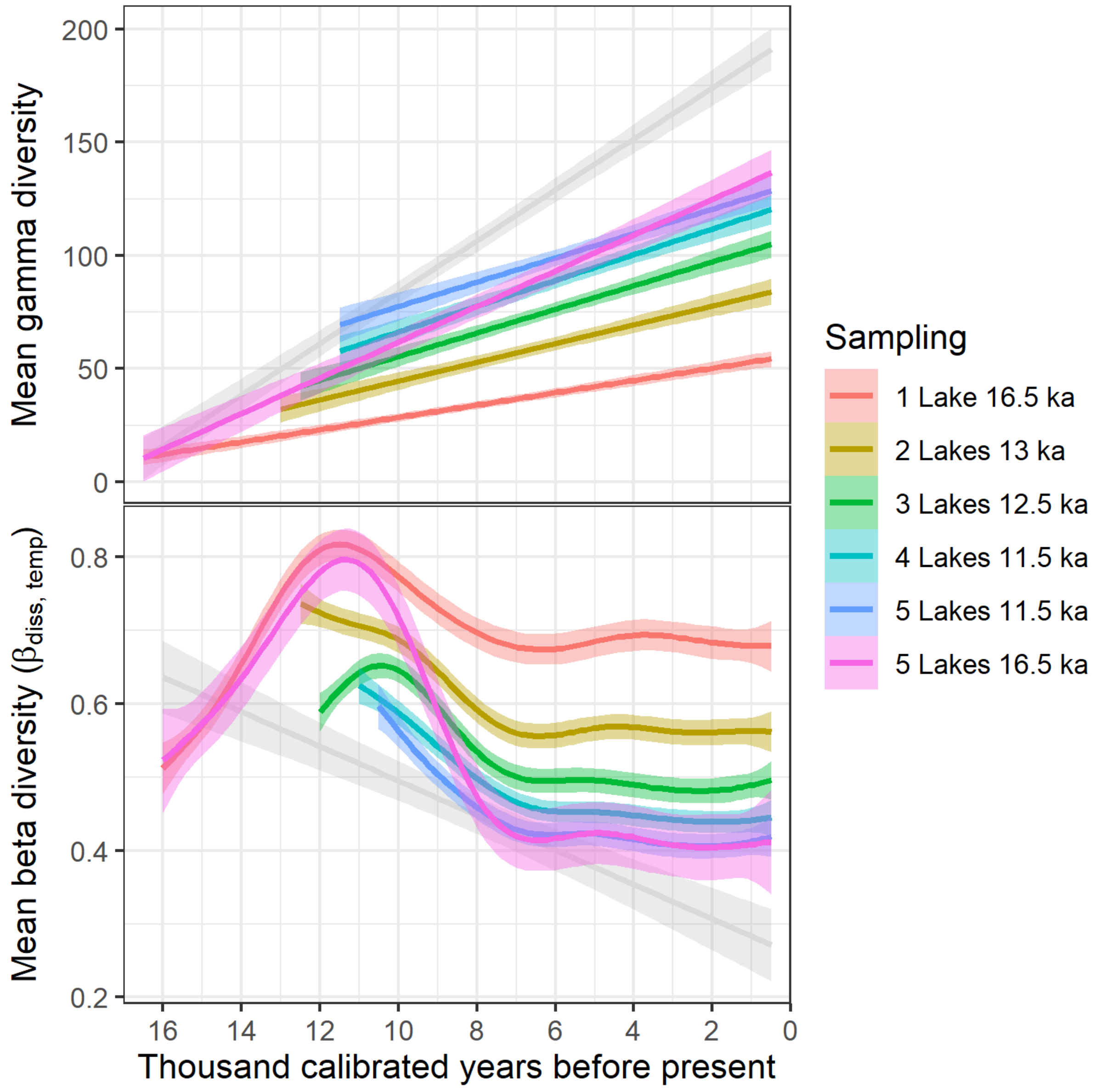

Fig. S2 Comparison of mean gamma diversity and mean temporal beta diversity developing over time based on 100 subsampling from the full dataset. Note that all 500-year intervals were retained while resampling the first data set, where each 500-year interval was represented by a single lake (and in some cases an interval was represented by a sample from a single lake). The second and third datasets covered the periods 13-0.5 ka and 12.5-0.5 ka, respectively, with each 500-year interval representing 2 and 3 lakes respectively. The fourth dataset included 4 lakes per interval from 11.5-0.5 ka, excluding the 10 ka interval which did not have a sufficient number of lakes. The fifth dataset included 5 lakes per interval from 11.5-0.5 ka, excluding the 11 and 10 ka intervals. The sixth dataset included a single

lake or sample from 16.5-12 ka, 11 and 10 ka intervals, and 5 lakes in the remaining intervals making its temporal span similar to the full dataset. Note that estimates of the temporal beta diversity are provided between time intervals and hence estimated values have a later starting time compared to the gamma diversity. The grey lines and shadings are based on a full dataset (and correspond to results presented in the main manuscript). The gamma and beta diversity trajectories are based on linear and generalized additive models respectively.

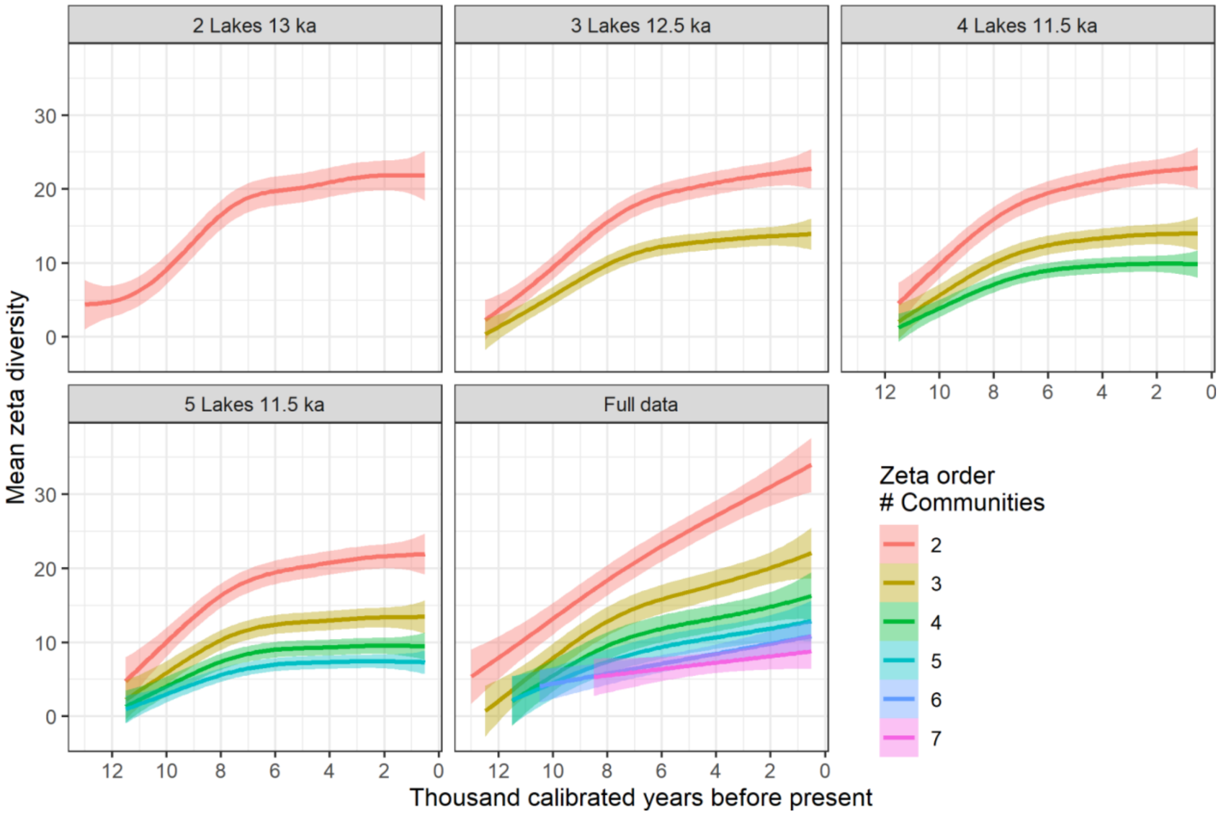

Fig. S3 Mean zeta diversity developing over time based on 100 subsampling from the full dataset. Note that all 500-year bins with minimum two lakes were selected while subsampling followed by an increase in the number of lakes within each bin wherever possible (see Fig. S2 above). In some instances, for example while subsampling 5 lakes within each 500-year bin, only data from up to 11.5 ka could be included and 10 and 11 ka intervals were removed to maximize the inclusion of lakes (see Fig. S2 above).

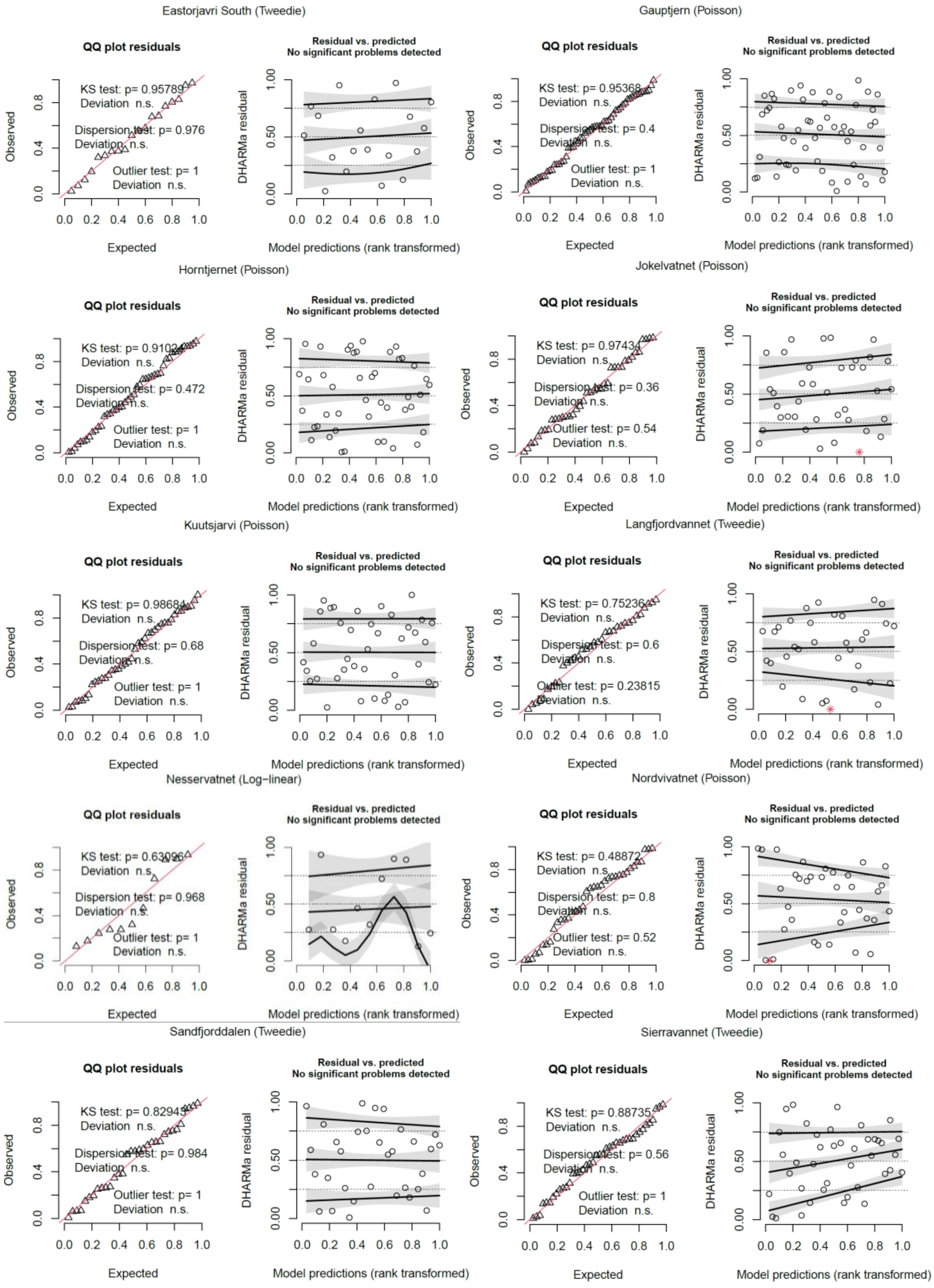

Fig. S4 Diagnostic plots for the GAM models used to evaluate alpha diversity (taxonomic richness) patterns through time in different lakes. The log-linear model excluding an outlier better fits the data than other models in Nesservannet.

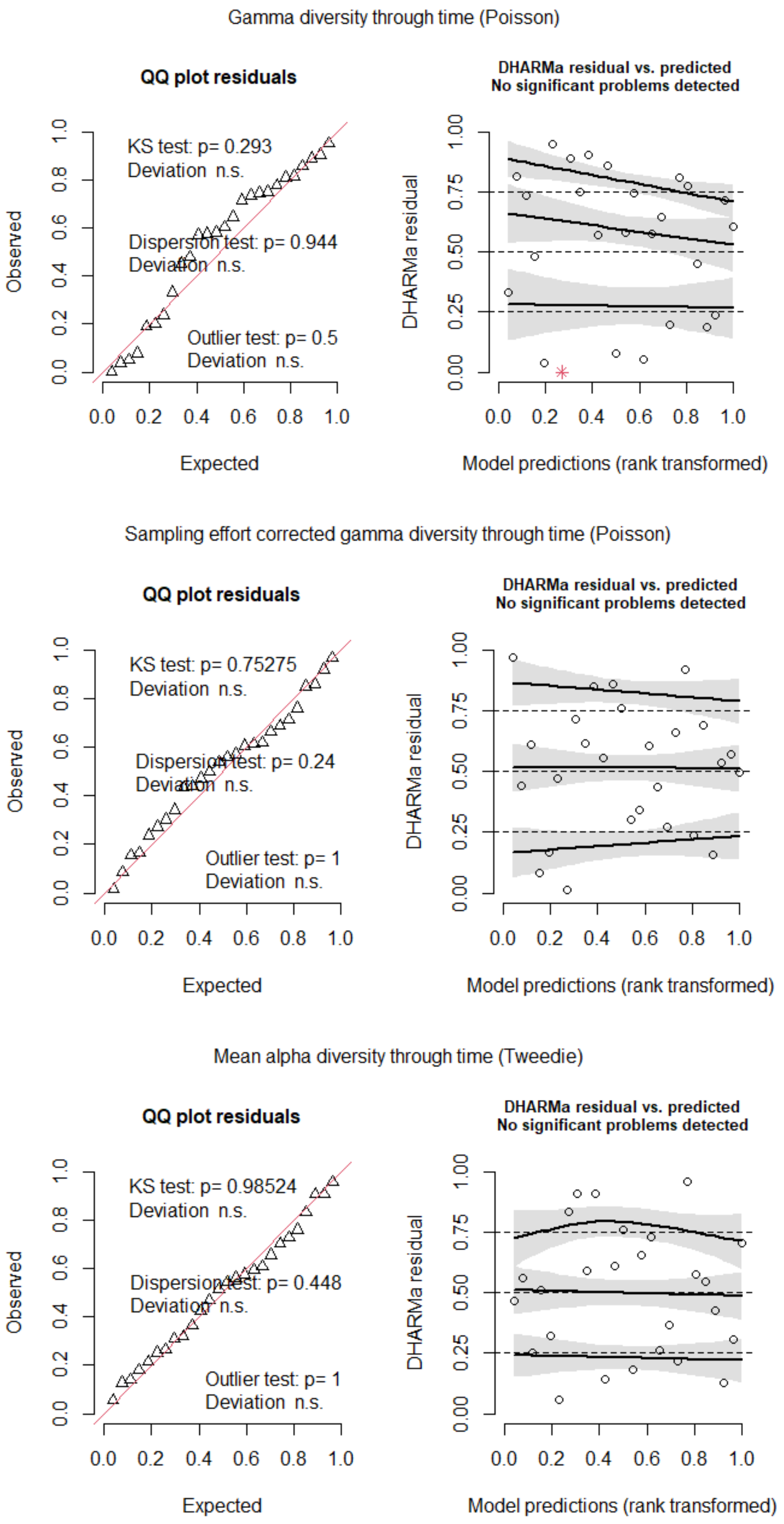

Fig. S5 Diagnostic plots for a *Poisson* (top and middle) and tweedie GAM (bottom) models used to evaluate the trend of gamma and sampling adjusted gamma (based on 3 lakes), and mean alpha diversity through time at 500-year intervals respectively.

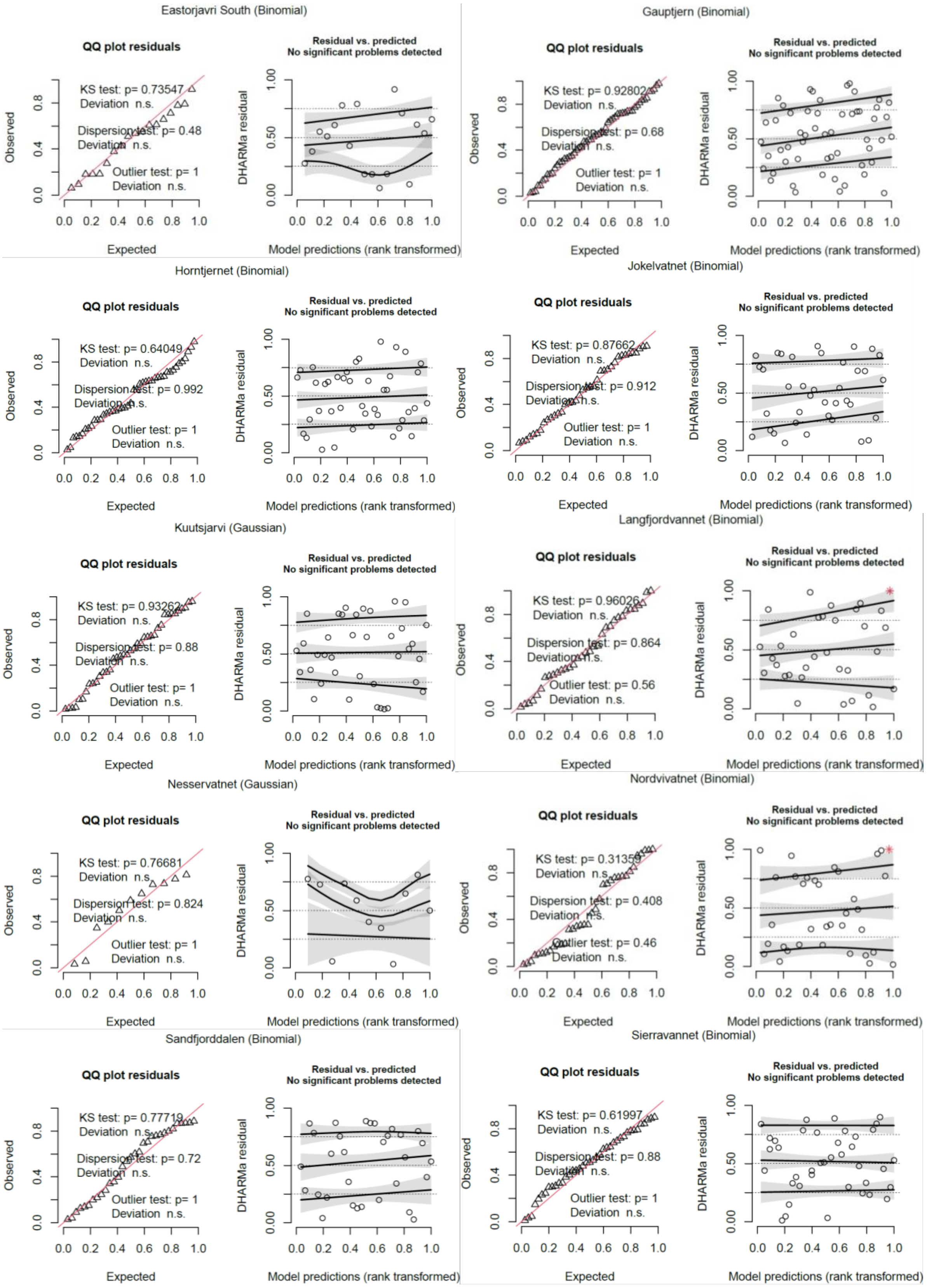

Fig. S6 Diagnostic plots for the GAM models used to evaluate species exchange ratio patterns through time in different lakes. The gaussian distribution indicated a better fit of the models in Kuutsjarvi and Nesservannet than binomial distribution.

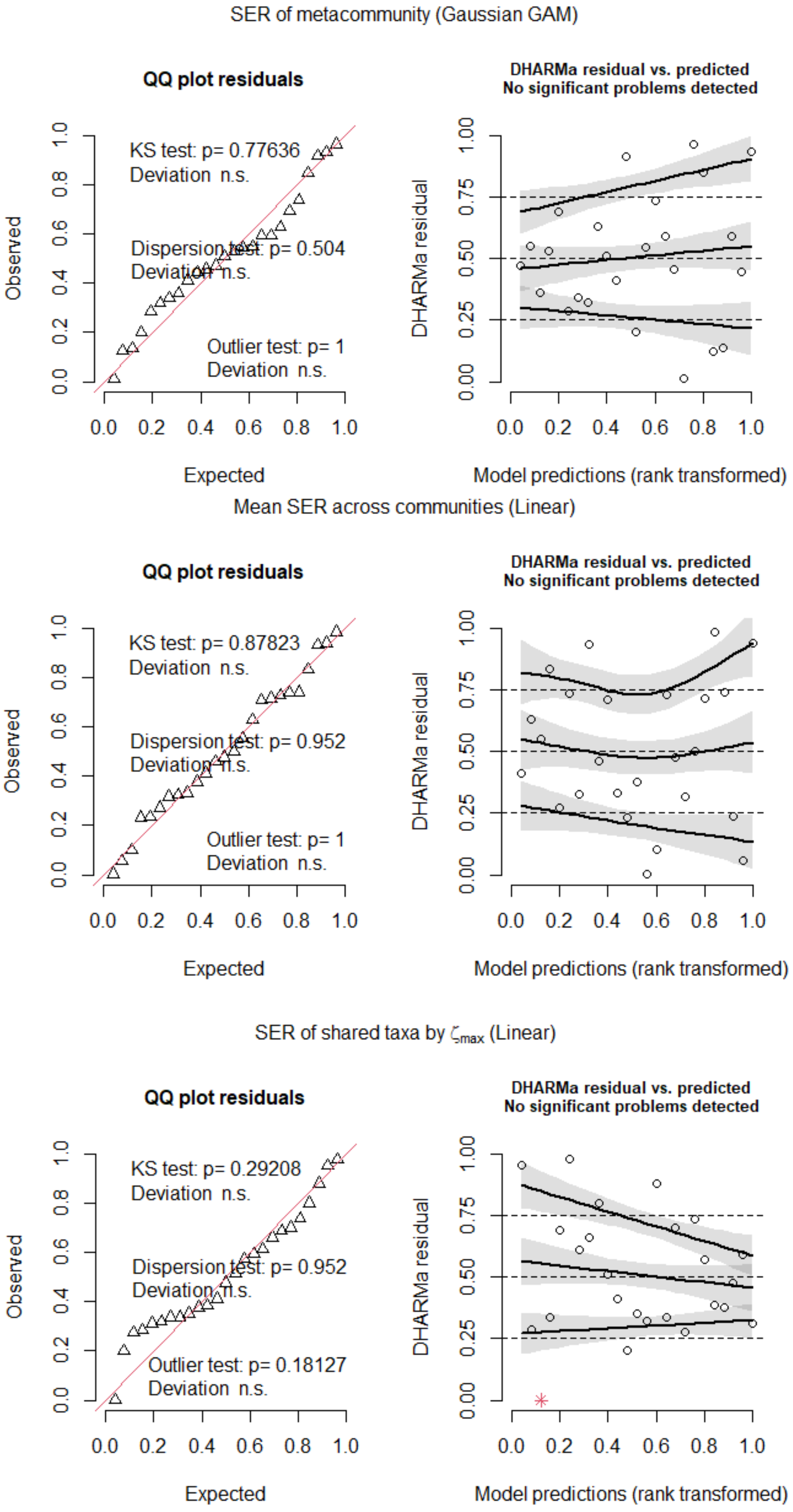

Fig. S7 Diagnostic plots for a Gaussian GAM evaluating temporal beta diversity (species exchange ratio, SER) of the metacommunity (top), and linear models for mean temporal beta diversity across communities (middle) and shared taxa (bottom) through 500-year intervals.

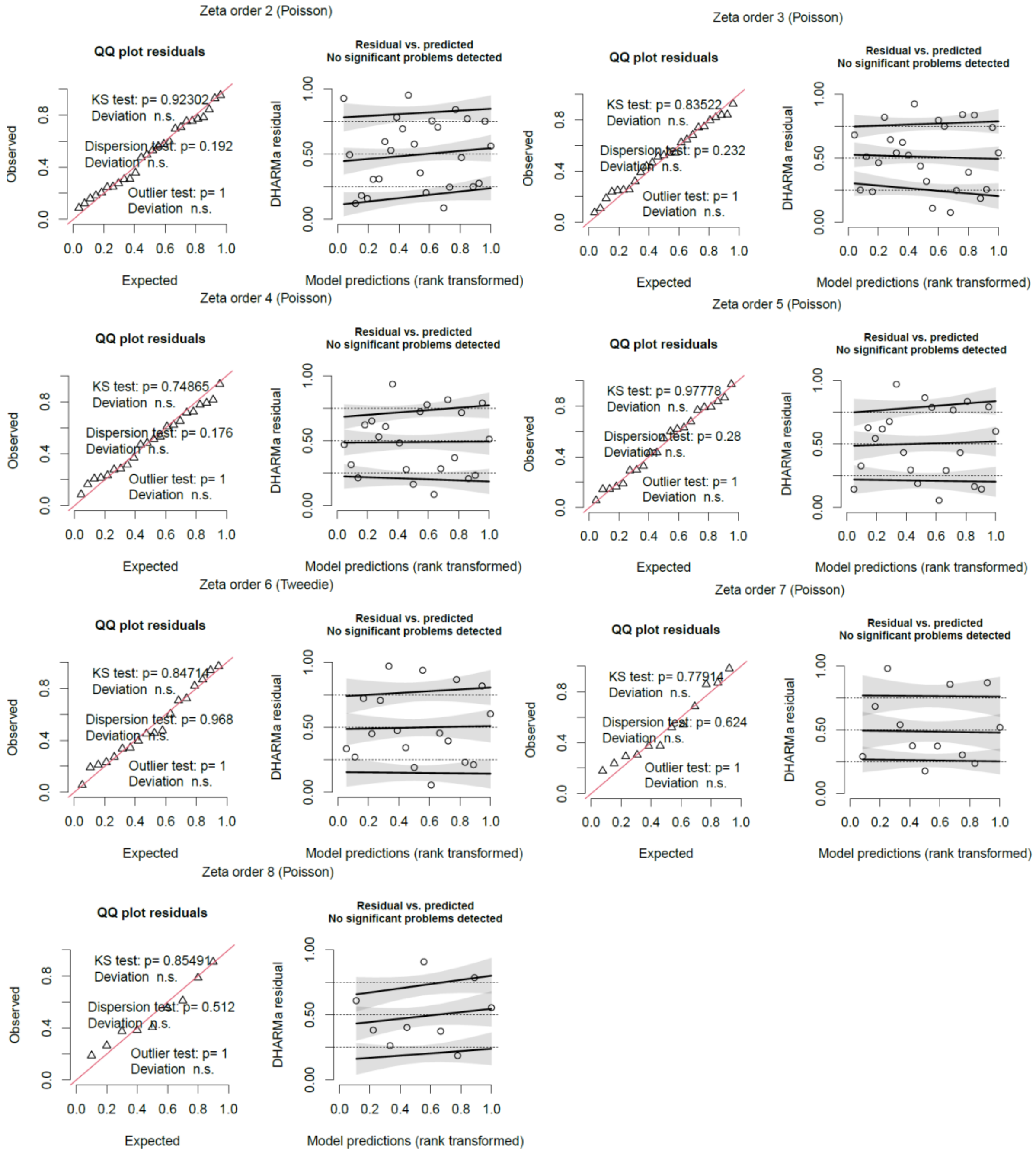

Fig. S8 Diagnostic plots for the GAM models used to evaluate zeta diversity patterns through time in 10 lake catchments at 500-year intervals.

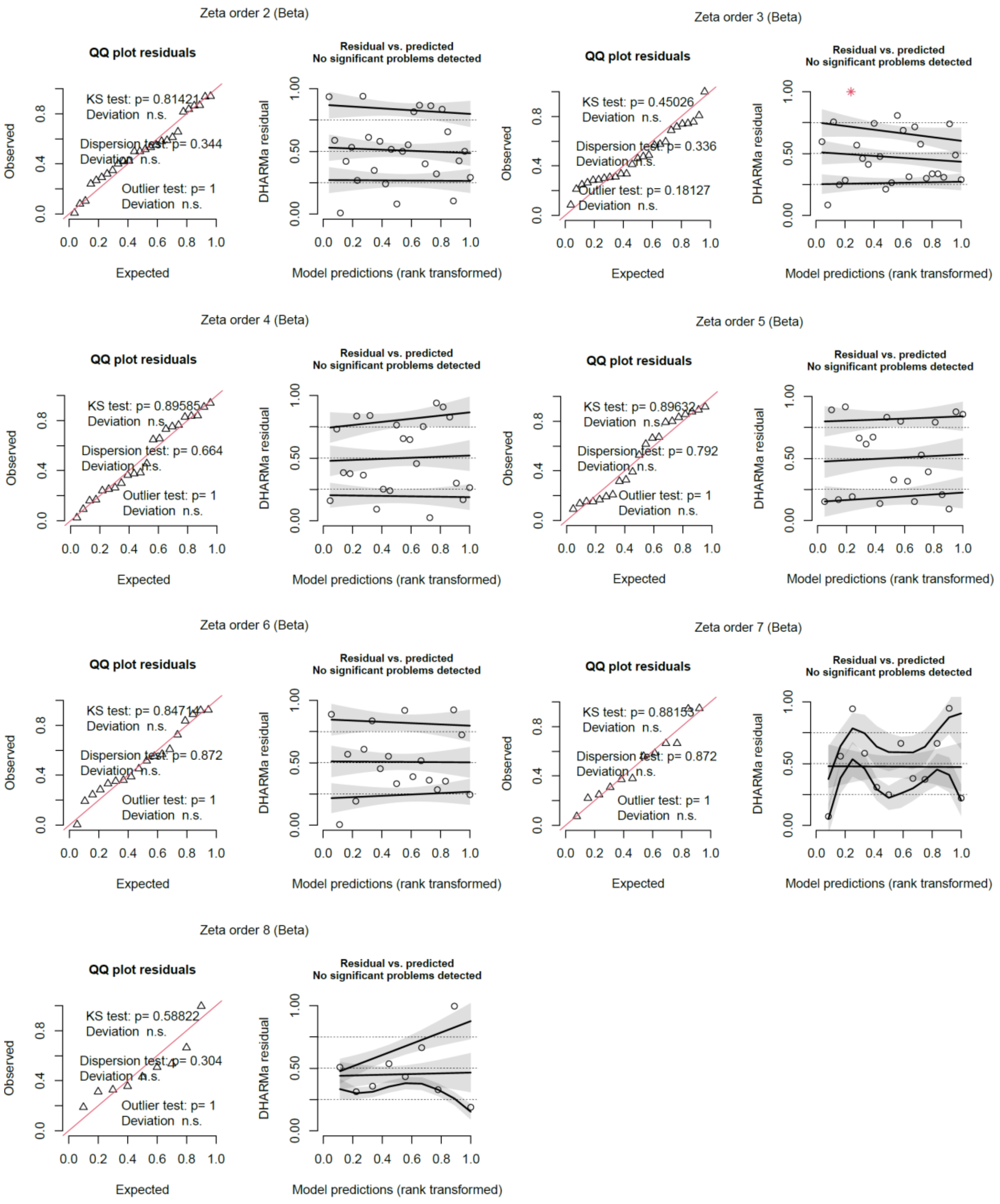

Fig. S9 Diagnostic plots for the GAM models used to evaluate zeta ratio patterns through time in 10 lake catchments at 500-year intervals.

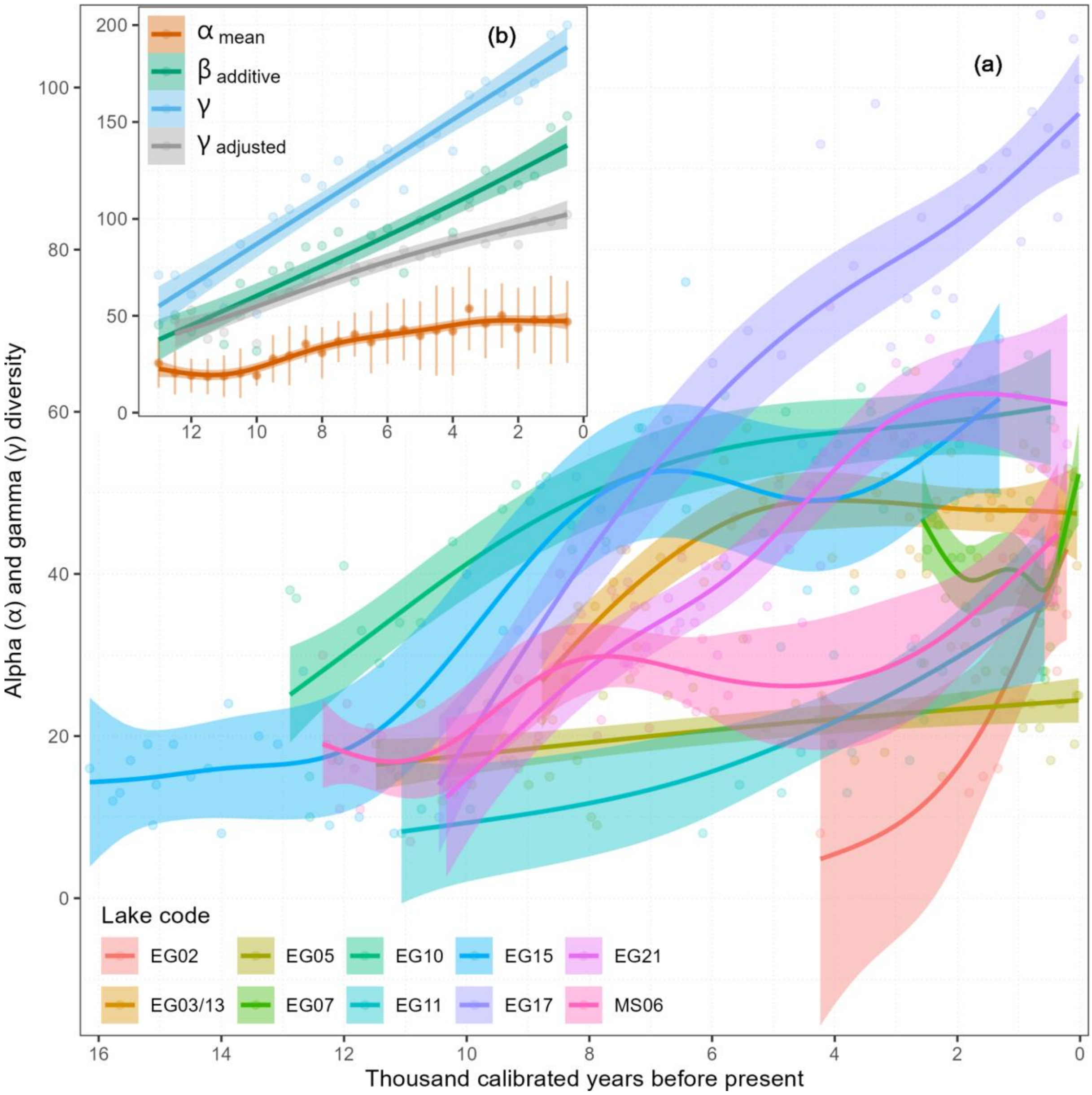

Fig. S10 Alpha, beta and gamma diversity during the Holocene. (a) Taxonomic richness of terrestrial plants (alpha diversity) in single catchment communities over time, and (b) the average alpha (±1SD), beta (Whitaker's additive beta diversity), gamma, and sampling adjusted gamma diversity across the metacommunity over time. The metacommunity estimates are based on sediment samples binned at 500-year intervals from minimum two lakes. The sampling adjusted gamma was estimated using three lakes per 500-year interval for the last 12.5 ka after 100 subsampling iterations. Shadings indicate 95% confidence intervals of the fitted models. See Fig. 2 for lake names.

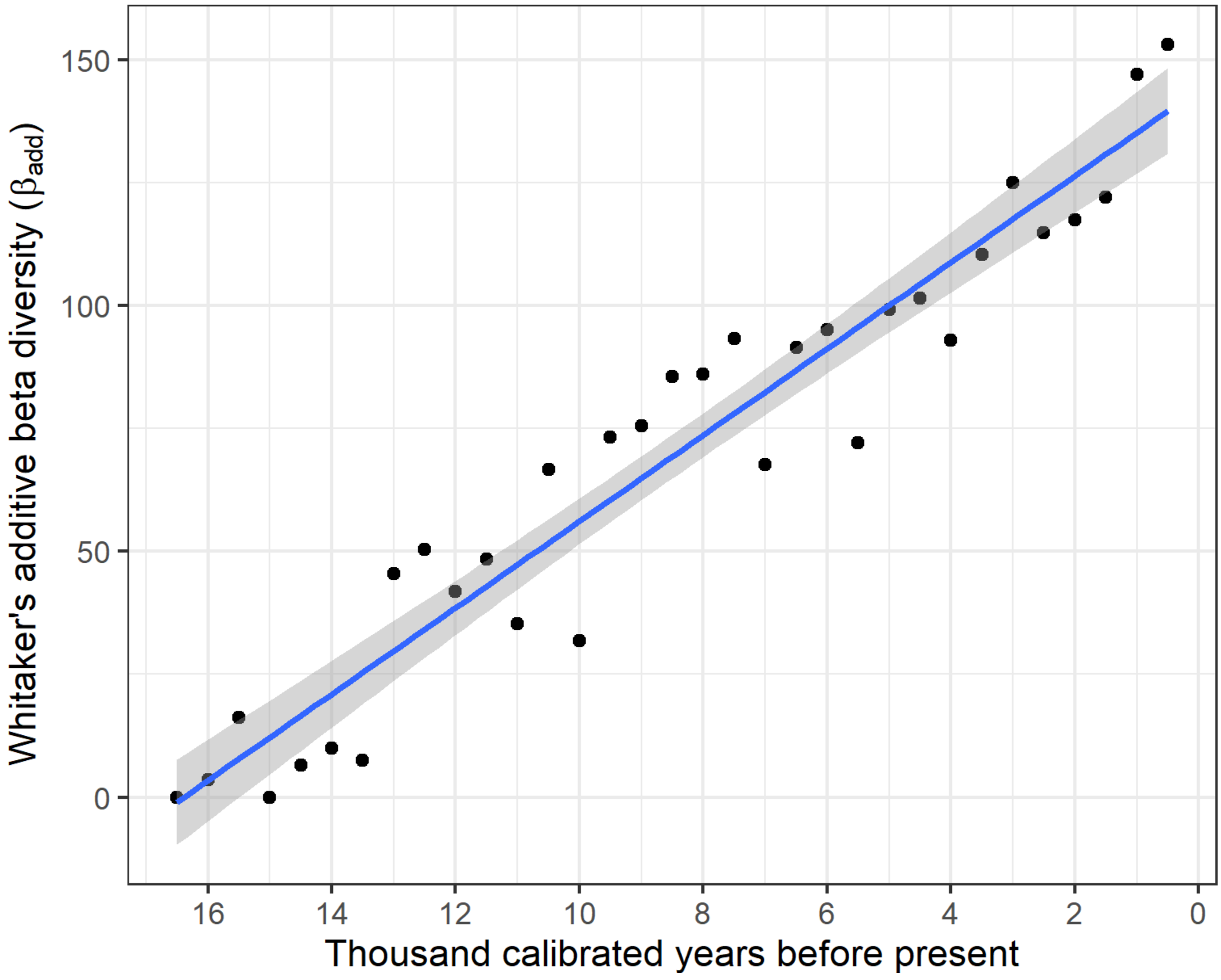

Fig. S11 Whitaker's additive beta diversity ($\beta_{add} = \gamma - \bar{\alpha}$) across the metacommunity over time. The metacommunity estimates are based on sediment samples binned at each 500-year interval. Note that intervals older than 13 ka are represented by a single lake. Shading indicates 95% confidence intervals of the fitted linear model.

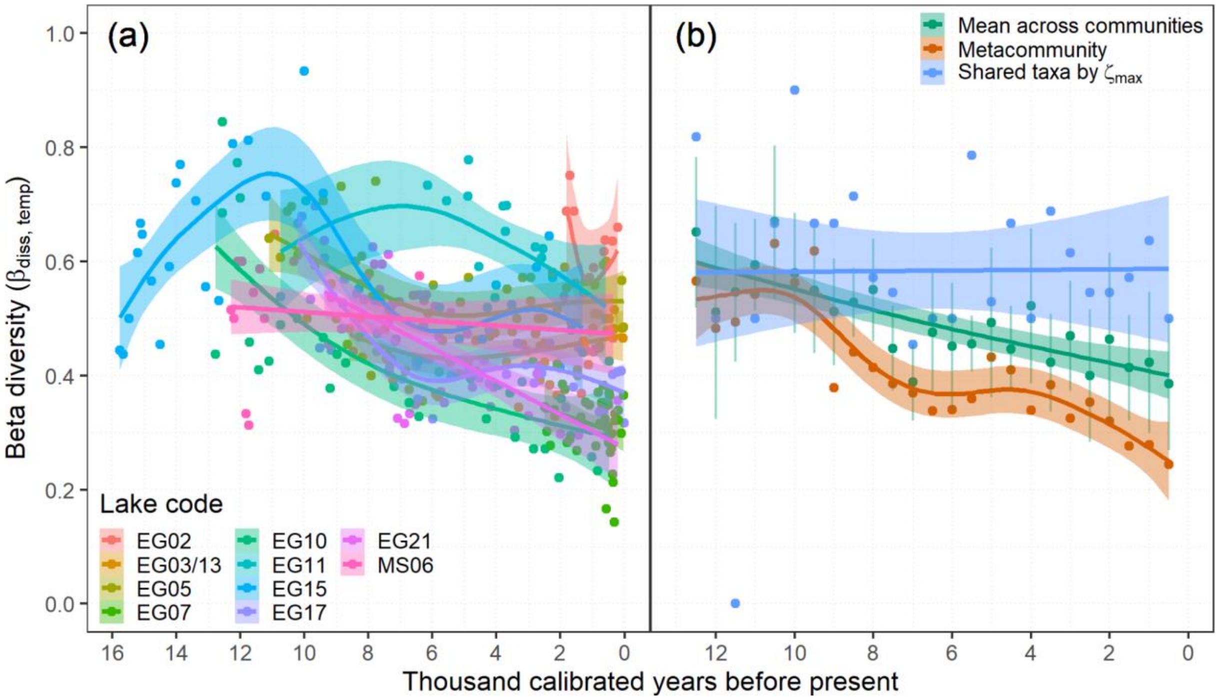

Fig. S12 Temporal beta diversity during the Holocene. The terrestrial plant taxa turnover (Jaccard's dissimilarity) between successive samples over time in (a) each of 10 communities, and (b) across the metacommunity including mean temporal beta diversity across local communities and the temporal beta diversity among the subset of shared taxa in the metacommunity. The subset of shared taxa applied here are those shared among the maximum number of communities at each time interval. The metacommunity estimates are based on sediment samples binned at 500-year intervals from minimum two lakes. Shadings indicate 95% confidence intervals of the fitted models. See Fig. 2 for lake names.

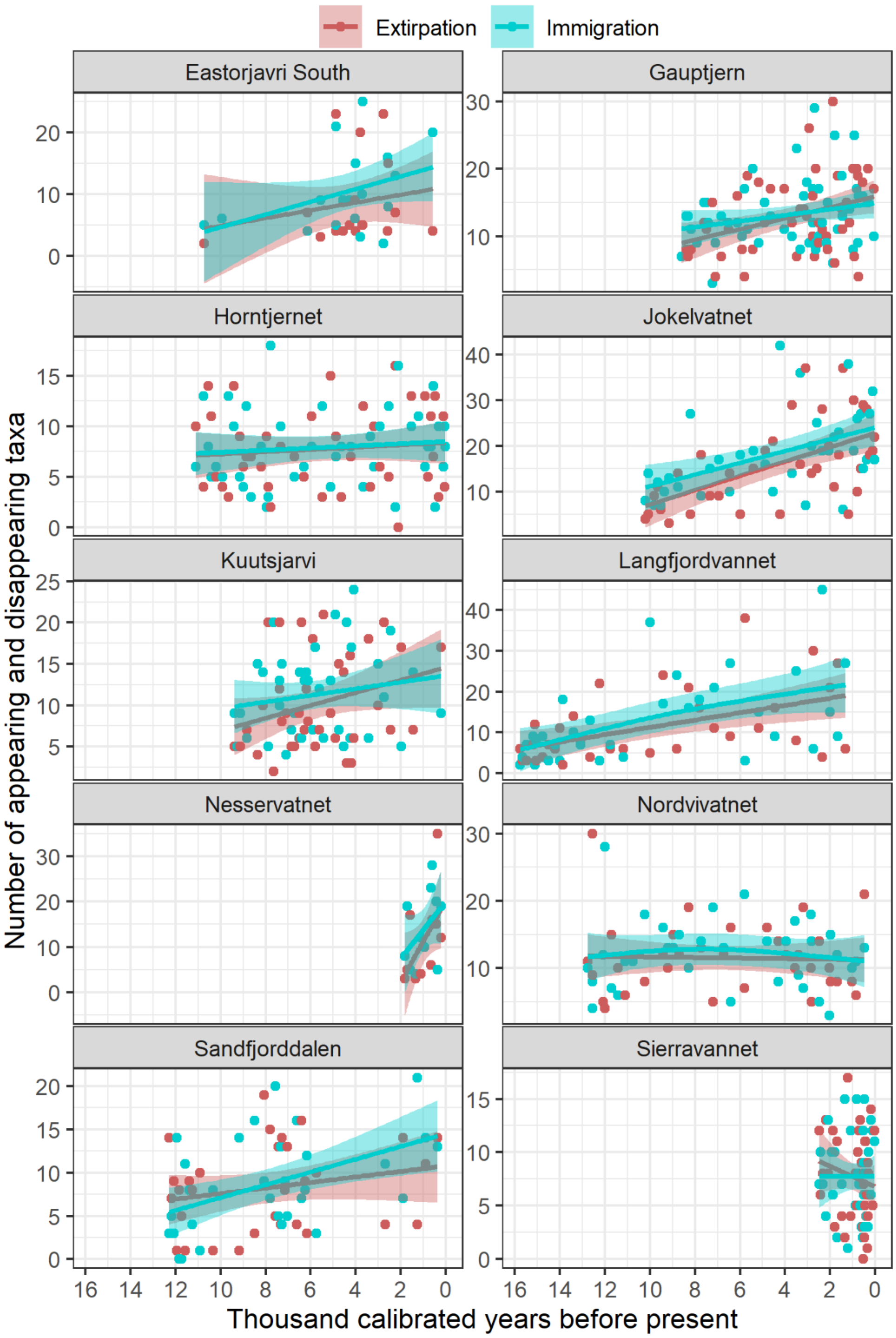

Fig. S13 Appearance and disappearance of taxa between consecutive samples within a catchment. Note the scale differences between y-axes.

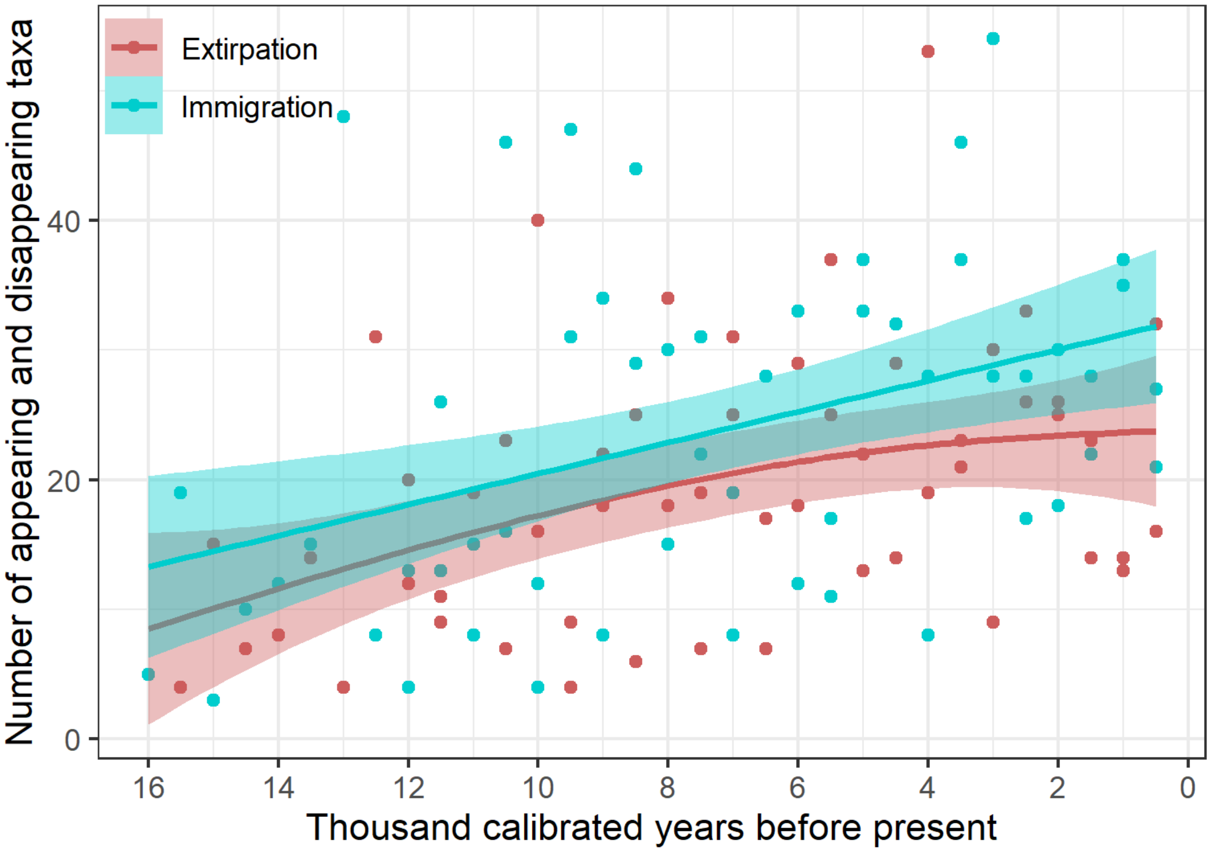

Fig. S14 Appearance and disappearance of taxa between consecutive 500-year intervals in the meta communities. Note that intervals older than 13 ka are represented by a single lake.

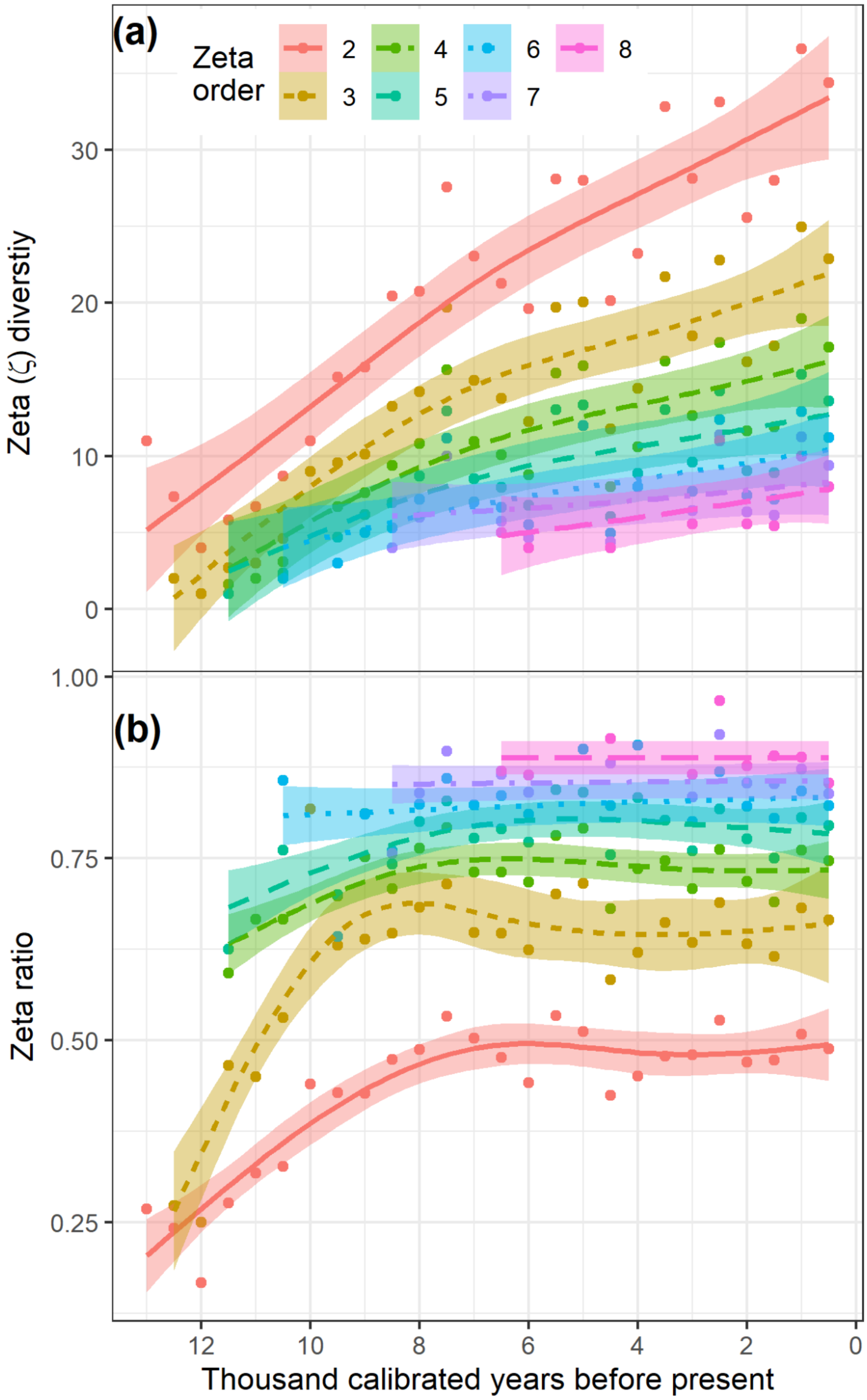

Fig. S15 Zeta diversity and zeta ratio during the Holocene. (a) Zeta diversity over time for 10 communities. Each line shows the average number of shared terrestrial plant taxa among two (zeta order of 2) and up to eight (zeta order of 8) communities. (b) Zeta ratio over time for 10 communities. The inset shows the retention rate, the relation between zeta ratio, and zeta order, at four distinct time-intervals. All estimates are based on sediment samples binned at 500-year intervals. Shadings indicate 95% confidence intervals of the fitted generalized additive models.

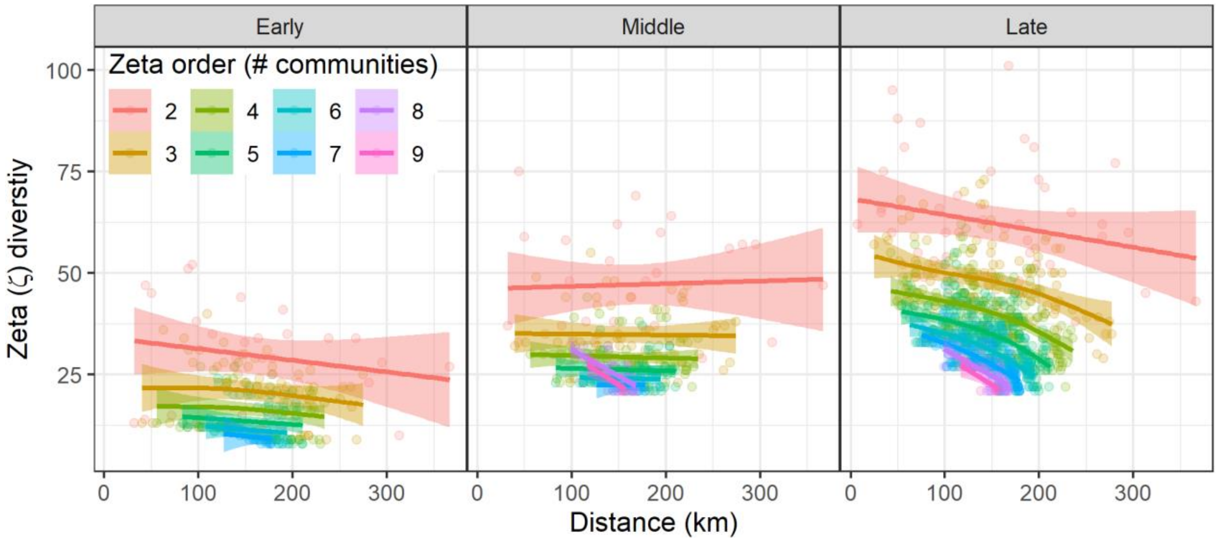

Fig. S16 Zeta diversity with distance during the Early, Middle, and Late Holocene. Each line shows the average number of shared terrestrial plant taxa in relation to distance between communities, among two (zeta order of 2) and up to nine (zeta order of 9) communities. Shadings indicate 95% confidence intervals of the fitted generalized additive models.

Supplementary tables

Table S1 Lakes metadata including terrestrial plant data used to generate diversity metrics.

Table S2 Test statistics for generalized additive models (GAM) used to evaluate temporal patterns of alpha diversity. Alpha diversity (taxonomic richness) was treated as the response and median calibrated sample age as the predictor variables. P-values of statistically significant smooth terms are indicated in the bold. edf: effective degrees of freedom, Chi.sq: test statistics based on chi-square test, Dev.exp: deviance explained, adj.R.sq: adjusted R-square

Table S3 Test statistics for generalized additive models (GAM) used to evaluate temporal patterns of beta diversity measured as species exchange ratio (SER). SER was treated as the response and median calibrated sample age as the predictor variables. P-values of statistically significant smooth terms are indicated in the bold. edf: effective degrees of freedom, Chi.sq: test statistics based on chi-square test, Dev.exp: deviance explained, adj.R.sq: adjusted R-square

Table S4 Test statistics for generalized additive models (GAM) used to evaluate temporal patterns of zeta diversity. Zeta diversity (the mean number of shared taxa among lakes within a 500-year interval) was treated as the response and 500-year intervals as the predictor variables. P-values of statistically significant smooth terms are indicated in the bold. edf: effective degrees of freedom, F: test statistics based on F test, Dev.exp: deviance explained, adj.R.sq: adjusted R-square

Table S5 Test statistics for generalized additive models (GAM) used to evaluate temporal patterns of zeta ratio. Zeta ratio (ratio of zeta diversity between consecutive samples) was treated as the response and 500-year intervals as the predictor variables. P-values of statistically significant smooth terms are indicated in the bold. edf: effective degrees of freedom, F: test statistics based on F test, Dev.exp: deviance explained, adj.R.sq: adjusted R-square

Table S6 Frequency of plant taxa in 500-year time intervals (N=169) and lakes (N=10)

Table S7 Test statistics for generalized additive models (GAM) used to evaluate zeta diversity patterns with distance among communities in each of the three Holocene periods.

Zeta diversity (the mean number of shared taxa among lakes within a 500-year interval) was treated as the response and Euclidean distance between lakes as the predictor variables for each of the periods. P-values of statistically significant smooth terms are indicated in the bold. Unreliable models, as indicated by residual plots (distribution tested: Linear, response transformed (log, logit, square-root) linear, Poisson, Negative binomial, Tweedie), either due to too few unique points or model misfit are indicated in red. edf: effective degrees of freedom, F: test statistics based on F test, Dev.exp: deviance explained, adj.R.sq: adjusted R-square

References

Hillebrand, H., Blasius, B., Borer, E.T., Chase, J.M., Downing, J.A., Eriksson, B.K., *et al.* (2018). Biodiversity change is uncoupled from species richness trends: Consequences for conservation and monitoring. *J. Appl. Ecol.*, 55, 169–184.

Koleff, P., Gaston, K.J. & Lennon, J.J. (2003). Measuring beta diversity for presence–absence data. *J. Anim. Ecol.*, 72, 367–382.

Lande, R. (1996). Statistics and partitioning of species diversity, and similarity among multiple communities. *Oikos*, 76, 5.

Socolar, J.B., Gilroy, J.J., Kunin, W.E. & Edwards, D.P. (2016). How Should Beta-Diversity Inform Biodiversity Conservation? *Trends Ecol. Evol.*, 31, 67–80.
